# Machine learning–guided multimodal profiling defines perturbed immune states at the time of cancer diagnosis

**DOI:** 10.1093/bib/bbag320

**Published:** 2026-06-17

**Authors:** Peggy Berlin, Amin Mirzaei, Felix Steinbeck, Martin Becker, Brigitte Müller-Hilke, Wendy Bergmann-Ewert, Daniel Dubinski, Thomas M Freiman, Daniel Strüder, Theresa Momper, Annabell Wolff, Philipp Kaps, Julia Henne, Clemens Schafmayer, Michael Linnebacher, Charlotte Wagner, Karen Rischmüller, Martin Philipp, Georg Lamprecht, Paul Meissner, Karoline Schulz, Christian Junghanss, Bernd Kreikemeyer, Sonja Oehmcke-Hecht, Claudia Maletzki

**Affiliations:** Department Internal Medicine, Division of Gastroenterology, Hepatology and Nutritional Medicine, Rostock University Medical Center, Ernst-Heydemann-Str. 6, 18057 Rostock, Germany; Department of Computer Science and Electrical Engineering, University of Rostock, Albert-Einstein-Str. 22, 18059 Rostock, Germany; Core Facility for Cell Sorting and Cell Analysis, Rostock University Medical Center, Schillingallee 70, 18057 Rostock, Germany; Department of Computer Science and Electrical Engineering, University of Rostock, Albert-Einstein-Str. 22, 18059 Rostock, Germany; Department of Mathematics and Computer Science, Marburg University, Hans-Meerwein-Straße 6, 35043 Marburg, Germany; Core Facility for Cell Sorting and Cell Analysis, Rostock University Medical Center, Schillingallee 70, 18057 Rostock, Germany; Core Facility for Cell Sorting and Cell Analysis, Rostock University Medical Center, Schillingallee 70, 18057 Rostock, Germany; Department of Neurosurgery, Rostock University Medical Center, University of Rostock, Schillingallee 35, 18057 Rostock, Germany; Department of Neurosurgery, Rostock University Medical Center, University of Rostock, Schillingallee 35, 18057 Rostock, Germany; Department of Otorhinolaryngology, Head and Neck Surgery “Otto Koerner”, Rostock University Medical Center, Doberaner Str. 137, 18057 Rostock, Germany; Department of Otorhinolaryngology, Head and Neck Surgery “Otto Koerner”, Rostock University Medical Center, Doberaner Str. 137, 18057 Rostock, Germany; Department of Internal Medicine—Clinic and Polyclinic for Hematology, Hemostaseology, Oncology, Stem Cell Therapy and Palliative Medicine, Rostock University Medical Center, Ernst-Heydemann-Straße 6, 18057 Rostock, Germany; Department of Internal Medicine—Clinic and Polyclinic for Hematology, Hemostaseology, Oncology, Stem Cell Therapy and Palliative Medicine, Rostock University Medical Center, Ernst-Heydemann-Straße 6, 18057 Rostock, Germany; Department of Operative Medicine, Clinic of General, Visceral, Thorax, Vascular and Transplantation Surgery, Rostock University Medical Center, Schillingallee 35, 18057 Rostock, Germany; Department of Operative Medicine, Clinic of General, Visceral, Thorax, Vascular and Transplantation Surgery, Rostock University Medical Center, Schillingallee 35, 18057 Rostock, Germany; Department of Operative Medicine, Clinic of General, Visceral, Thorax, Vascular and Transplantation Surgery, Rostock University Medical Center, Schillingallee 35, 18057 Rostock, Germany; Department of Internal Medicine—Clinic and Polyclinic for Hematology, Hemostaseology, Oncology, Stem Cell Therapy and Palliative Medicine, Rostock University Medical Center, Ernst-Heydemann-Straße 6, 18057 Rostock, Germany; Department Internal Medicine, Division of Gastroenterology, Hepatology and Nutritional Medicine, Rostock University Medical Center, Ernst-Heydemann-Str. 6, 18057 Rostock, Germany; Department Internal Medicine, Division of Gastroenterology, Hepatology and Nutritional Medicine, Rostock University Medical Center, Ernst-Heydemann-Str. 6, 18057 Rostock, Germany; Department Internal Medicine, Division of Gastroenterology, Hepatology and Nutritional Medicine, Rostock University Medical Center, Ernst-Heydemann-Str. 6, 18057 Rostock, Germany; Department of Internal Medicine—Clinic and Polyclinic for Hematology, Hemostaseology, Oncology, Stem Cell Therapy and Palliative Medicine, Rostock University Medical Center, Ernst-Heydemann-Straße 6, 18057 Rostock, Germany; Department of Medical Biology and Electron Microscopy Center, Rostock University Medical Center, Strempelstraße 14, 18057 Rostock, Germany; Department of Internal Medicine—Clinic and Polyclinic for Hematology, Hemostaseology, Oncology, Stem Cell Therapy and Palliative Medicine, Rostock University Medical Center, Ernst-Heydemann-Straße 6, 18057 Rostock, Germany; Institute of Medical Microbiology, Virology and Hygiene, University Medical Center Rostock, Schillingallee 70, 18057 Rostock, Germany; Institute of Medical Microbiology, Virology and Hygiene, University Medical Center Rostock, Schillingallee 70, 18057 Rostock, Germany; Department of Internal Medicine—Clinic and Polyclinic for Hematology, Hemostaseology, Oncology, Stem Cell Therapy and Palliative Medicine, Rostock University Medical Center, Ernst-Heydemann-Straße 6, 18057 Rostock, Germany

**Keywords:** multimodal data integration, explainable machine learning

## Abstract

Altered immune states at the time of cancer diagnosis remain insufficiently characterized. Although circulating immune biomarkers offer a promising, non-invasive way of analysing systemic tumour–host interactions, their potential remains poorly defined. Here, we present an integrated multi-omics analysis of peripheral blood mononuclear cells from treatment-naïve cancer patients, minimizing confounding by therapy-induced immune changes, combining immune phenotyping (flow cytometry, FC), multiplex cytokine profiling, and single-cell RNA sequencing (scRNA-seq). Compared with healthy donors, patients exhibited widespread immune dysregulation, including expansion of FOXP3^+^ regulatory T cells, depletion of CD16^+^CD11b^+^ monocytes and CD56^dim^ Natural killer (NK) cells, and elevated plasma IL-6 and IL-4 levels. scRNA-seq identified cancer-associated immune signatures, notably consistent upregulation of *THBS1* and *CH25H*, indicative of systemic imprinting by tumour-derived cues. We further developed machine learning-guided models integrating single-cell multi-omics data (sc-FC and scRNA-seq) to characterize cancer-associated immune patterning and cancer type–related signal structure, while providing biologically interpretable feature attribution across modalities. The models achieved robust classification performance within the cohort and revealed modality-spanning features linked to immune state alterations.

Together, these findings establish a framework for immune-based, multi-omics profiling of peripheral blood and provide a resource for discovering circulating cancer-associated immune signatures. This supports future development of immune-based diagnostics and disease monitoring approaches.

## Introduction

Immunological alterations at the time of cancer diagnosis are not well characterized. This is partly because most solid tumours only become symptomatic at advanced stages, and current screening tools are limited to a few types of cancer. Although conventional imaging modalities—including Magnetic Resonance Imaging and Positron Emission Tomography–Computed Tomography—remain essential for clinical management, they lack sensitivity needed for early or minimal residual disease detection. They also have limited ability to capture the spatial and functional heterogeneity of the tumour-immune microenvironment [1, 2]. In this context, circulating biomarkers have emerged as valuable, minimally invasive tools for cancer diagnosis, disease monitoring, and therapy guidance. Metrics like neutrophil-to-lymphocyte ratio have demonstrated prognostic utility [3–6], and circulating tumour DNA (ctDNA) analysis has transformed early detection and real-time surveillance strategies [7–9]. However, current liquid biopsy approaches predominantly focus on tumour-derived analytes. Therefore, they provide limited insight into the systemic immune perturbations that often precede detectable ctDNA release or radiologic progression. It is important to note that immune dysregulation itself carries diagnostic and prognostic information that existing tumour markers do not capture.

Cancer arises from dysregulation of key cellular pathways, including proliferation, apoptosis, metabolism, and immune function, with immune dysfunction and exhaustion playing central roles in progression [10–13]. Immunotherapeutic strategies seek to restore anti-tumour immunity and have become integral to modern oncology [14–19]. These advances underscore the importance of understanding systemic immune states as reflections of tumour–host interactions.

The state-of-the-art single-cell sequencing technology offers unprecedented resolution for profiling cellular heterogeneity and identifying rare immune subsets and cellular states [20–23]. For instance, peripheral expansion of immunosuppressive immature neutrophils has been observed in gastric cancer, whereas hepatocellular carcinoma is associated with attenuated Transforming growth factor (TGF)β signalling in tumour-infiltrating CD8^+^ T cells [22, 24]. Large-scale analyses of peripheral blood mononuclear cells (PBMCs) further revealed distinct immune cell patterns and activation states between cancer patients and healthy individuals [25]. Accordingly, analytical tools for the in-depth investigation of tumour–immune interactions are becoming increasingly available, enabling a deeper understanding of the underlying dynamic changes [20, 26]. Despite this growing number of available sc-RNA-seq datasets, many studies lack comprehensive clinico-pathological annotation and complementary immunophenotypic or cytokine profiling. This limits the development of robust, generalizable predictive models. Moreover, few studies explore systematic integration of multiple single cell–resolved immune modalities. However, integrating multimodal data together with detailed clinical metadata is essential to advance machine learning algorithms that can interrogate complex immune states and explore their associations with disease phenotypes. More rigorous data curation and harmonization are therefore required to enhance diagnostic consistency and clinical applicability.

Using an integrative multi-omics approach combining laboratory data and scRNA-seq from treatment-naïve solid tumour patients, we identified systemic immune dysregulation marked by elevated T helper cell 2 (Th2)cytokines. Next, we trained a deep neural network to recognize cancer-associated immune states and transcriptional signatures. These integrated analyses establish a multimodal framework for exploring alterations in circulating immune cells in cancer and for generating biologically interpretable hypotheses regarding immune-associated disease patterns.

## Material and methods

### Patient recruitment and ethical statement

**Figure 1:**
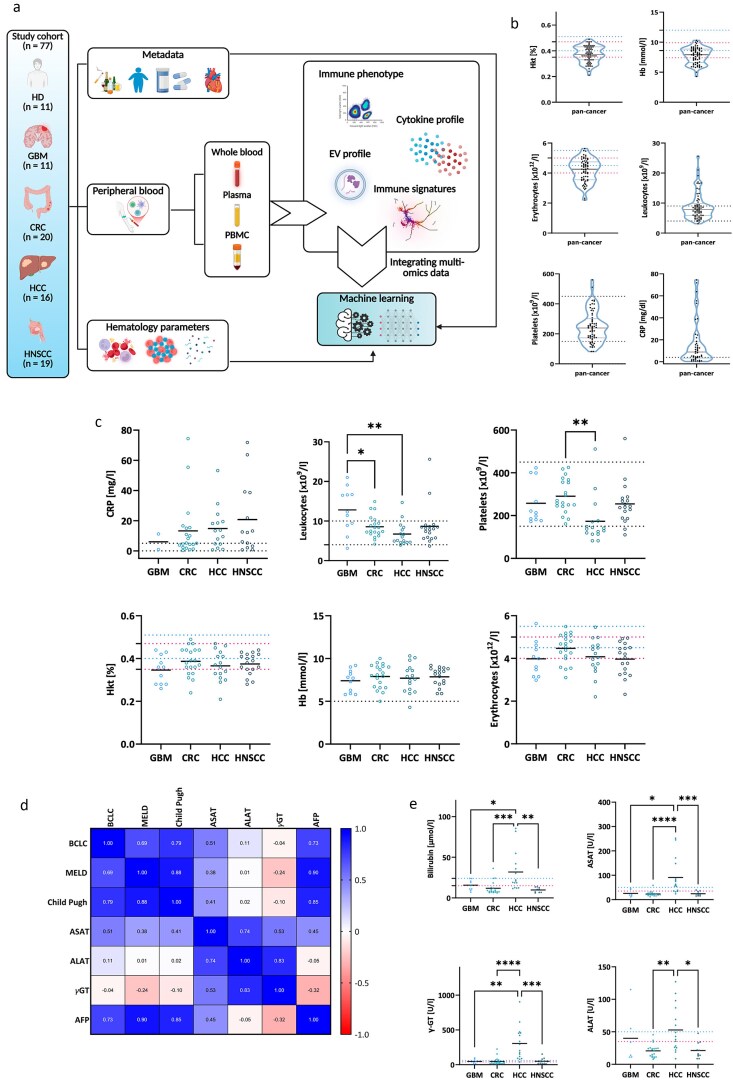
Experimental workflow and blood parameters of oncological patients. (a) Study design. (b, c, e) Peripheral blood was collected from HD—healthy donors (*n* = 11) and patients with GBM—glioblastoma (*n* = 11), CRC—colorectal cancer (*n* = 20), HCC—hepatocellular cancer (*n* = 16), and HNSCC—head and neck squamous cell carcinoma (*n* = 16). Patients’ blood was analysed in the clinical chemistry and laboratory medicine. Blood cells and liver enzymes were quantified. Data are either shown as (b) violin plots (+ individual points) for the entire cancer cohort or (c, e) stratified by cancer type depicted as scatter plot + median. One-way ANOVA (Tukey’s multiple comparisons test), ^*^P < .05; ^**^P < .01; ^***^P < .001; ^****^P < .0001. (b) (Gender-specific) reference values (i.e. blue for male; pink for female) are given as dotted lines. *n* = 45–65 data/parameter. (d) Correlation matrix showing the association between clinic-pathological and blood-based parameters relevant to HCC. Pearson *r* analysis.

This cross-sectional study included cancer patients (*n* = 66) and healthy volunteers (*n* = 11). Written informed consent was obtained from all study participants. All procedures were approved by the local Ethics Committee (Rostock University Medical Center, Ethics Registration ID: A2018-0167, A2024-0112, A2022-0064). Patients were recruited between March 2023 and August 2024. Patients with cancer were diagnosed as follows: WHO grade 4 glioblastoma (*n* = 11), colorectal cancer (*n* = 20), hepatocellular carcinoma (*n* = 16), and head and neck squamous cell carcinoma (*n* = 19). Clinical patient information, including noxae (smoking, alcohol) were recorded (Table 1). CRC samples were obtained as part of the HROC biobanking program (Supplemental Table 2). Blood samples were taken at initial diagnosis, i.e. before surgery or systemic treatment initiation (=treatment-naïve). Blood was collected and processed immediately (Fig. 1).

**Table 1:** Clinico-pathological data of the pan-cancer patient cohort (total) as well as the individual cancer cohorts—glioblastoma (GBM), colorectal cancer (CRC), hepatocellular carcinoma (HCC), and head and neck squamous cell carcinoma (HNSCC).

Number [*n*]	Total	GBM	CRC	HCC	HNSCC
	66	11	20	16	19
Age [years] Average Median Range	65.7 65.3 (33.5–85.6)	68.7 72.5	64.1 64.9	66.9 68.0	64.6 62.6
Gender [*n*] Female [%] Male [%]	21 [31.8] 45 [68.2]	6 [54.5] 5 [45.5]	6 [30.0] 14 [70.0]	4 [25.0] 12 [75.0]	5 [26.3] 14 [73.7]
Smoker [*n*] Yes [%] No [%]	19 [28.8] 47 [71.2]	2 [18.2] 9 [81.8]	2 [10] 18 [90]	5 [18.2] 11 [81.8]	10 [52.6] 9 [47.4]
Alcohol [*n*] Yes [%] No [%] Unknown [%]	18 [27.3] 26 [39.4] 22 [33.3]	0 [0.0] 11 [100.0] 0 [0.0]	0 [0.0] 20 [100.0] 0 [0.0]	8 [50.0] 2 [12.5] 6 [37.5]	10 [52.6] 4 [21.1] 5 [26.3]
History of cancer [*n*]^*^ Yes [%] No [%] Unknown [%]	16 [24.2] 46 [69.7] 4 [6.1]	4 [36.4] 6 [54.5] 1 [9.1]	3 [15.0] 15 [75.0] 2 [10.0]	4 [25.0] 12 [75.0] 0 [0.0]	5 [26.3] 13 [68.4] 1 [5.3]
Obesity [*n*]^a^ Yes [%] No [%] Unknown [%]	22 [33.3] 22 [33.3] 22 [33.3]	1 [9.1] 3 [27.3] 7 [63.6]	11 [55.0] 9 [45.0] 0 [0.0]	5 [31.2] 1 [6.3] 10 [62.5]	5 [26.3] 9 [47.4] 5 [26.3]
Prior VTE/PVT^*^ [*n*] Yes [%] No [%]	9 [13.6] 57 [86.4]	3 [27.3] 8 [72.7]	1 [5.0] 19 [95.0]	3^*^ [18.8] 13 [81.2]	2 [10.5] 17 [89.5]
CVR [*n*] Yes [%] No [%]	32 [48.5] 34 [51.5]	5 [45.5] 6 [54.5]	15 [75.0] 5 [25.0]	7 [43.7] 9 [56.3]	5 [26.3] 14 [73.7]
CVD [*n*] Yes [%] No [%]	44 [66.7] 22 [33.3]	9 [81.8] 2 [18.2]	15 [75.0] 5 [25.0]	9 [56.3] 7 [43.7]	11 [59.9] 8 [42.1]

^a^BMI ≥ 25 kg/m^2^ (not further specified). ^*^Including benign neoplasms (1× oncocytoma, 1× pituitary adenoma, 1× prostatic hyperplasia, 1× liver haemangioma). VTE, venous thromboembolism; PVT, portal vein thrombosis; CVR, cardiovascular risk; CVD, cardiovascular disease

### Clinical chemistry and blood processing for laboratory analyses

Routine laboratory parameters were obtained from blood samples processed as follows (Fig. 1): (I) two 0.5-ml aliquots of whole blood were centrifuged (1000*×g*, 10 min), and the pellets were resuspended in freezing medium (FCS + 10% DMSO) and stored in liquid nitrogen. (II) The remaining blood was centrifuged (900*×g*, 10 min) to collect plasma, which was (III) aliquoted (0.5 ml) and stored at −80 °C. (IV) The cellular fraction underwent density gradient centrifugation (1150*×g*, 15 min) to isolate PBMCs, which were counted and cryopreserved in liquid nitrogen.

### Extracellular vesicle isolation and nanoparticle tracking analysis

Extracellular vehicles (EVs) were isolated from 0.5 ml of preserved plasma by differential centrifugation. Plasma was diluted 1:2 with sterile PBS and centrifuged (16 000*×g*, 10 min, 4 °C) to remove debris and large particles. The supernatant was transferred to ultracentrifugation tubes, filled with PBS, and ultracentrifuged using a Beckman Coulter Optima XPN-80 with an SW40 Ti rotor (120 000*×g*, 120 min, 4 °C). Pelleted EVs were resuspended in PBS and analysed by Nanoparticle Tracking Analysis (NanoSight, NTA 3.3) to determine particle size and concentration. Each sample was recorded five times for 30 s, and mean values were calculated. Background particles were excluded by subtracting PBS controls. EV morphology was further examined by scanning electron microscopy using high resolution imaging on carbon-coated samples.

### Whole transcriptome single-cell sequencing (sc-RNA-seq)

PBMCs were thawed and counted, and 2 × 10^5^ cells were labelled using the BD Hu Single-Cell Multiplexing Kit according to the manufacturer’s protocol. Cells were stained with Calcein AM and DRAQ7, counted at the BD Rhapsody Scanner, and a suspension of 40 000 single cells prepared. Single-cell capture, bead-based mRNA isolation, and cDNA synthesis were performed using the BD Rhapsody Single-Cell Analysis System with the Enhanced Cartridge Reagent Kit. Reverse transcription was carried out with the BD Rhapsody cDNA Kit, followed by exonuclease I inactivation (80 °C, 20 min) and bead storage at 4 °C. Random priming, extension PCR, and index PCR were performed using the BD Rhapsody Whole Transcriptome Analysis Amplification Kit. Libraries were sequenced on a NovaSeq X Plus PE150-10B platform (Illumina) by Novogene GmbH (Martinsried, Munich, Germany).

### Sc-RNA-seq data analysis and quality control

Raw sc-RNA-seq reads were aligned to the human genome and annotated by cell type using the BD Rhapsody Sequence Analysis Pipeline 2.2.1. [27]. The standard preprocessing workflow for sc-RNA experiments and further analyses were conducted using the Seurat 5.1.0 package [28] in R version 4.4.1 [29]. As a first step, cells exhibiting either an excessive or insufficient number of unique RNA products were excluded, suggesting doublets/multiplets and low-quality cells. Secondly, cells expressing >25% of mitochondrial genes were considered either damaged or apoptotic and were excluded as well. A total of 111 141 putative cells could successfully be recovered from eight cartridges. Of those, 100 373 cells met the quality control criteria and were subjected to further analysis. Then, the raw count data were being normalized to counts-per-million reads, and highly variable features (genes) were identified. After scaling the data (*z*-score), dimensional reduction was performed, creating a UMAP for visualization purposes.

Differentially expressed genes between different tumour entities were determined for each cell type by Wilcoxon rank-sum test with a threshold of *P* < 10^−32^ and fold change >2. Highly significant genes are shown as volcano plots, heatmaps [30], bubble plots [31], and upset plots [32]. Furthermore, a KEGG pathways analysis for the DEGs was conducted using protti [33].

### Spectral flow cytometry

The differences in PBMCs between cancer patients and healthy donors were first analysed by spectral flow cytometry. An in-house multi-colour panel (Supplemental Table 5) was employed, and data from 73 samples (including 11 HD and 62 cancer patients) were included. Vitally stored blood samples were thawed, washed, and subjected to antibody staining. Additionally, the Human Th Cytokine Panel (12-plex) was used to quantify cytokine profiles in plasma samples.

The samples were analysed with the Cytek Aurora spectral flow cytometer, which is equipped with three lasers (violet (405 nm), blue (488 nm), and red (640 nm)). In total, 50 000 events were measured in a live gate. Data acquisition was done with the SpectroFlo Version 3.0 software.

### Development of linear and multimodal deep-learning models for cancer and healthy state prediction

To perform patient-level disease state prediction using sc-FC and sc-RNA-seq profiles, we designed a multimodal DL classifier consisting of two modality-specific encoders and a shared prediction head, trained separately for each binary classification task. sc-FC features were arcsinh-transformed (arcsinh(*x*/5)), and sc-RNA-seq features were taken from the precomputed highly variable gene matrix (sc-RNA-seq data analysis/quality control). The sc-FC and sc-RNA-seq branches were implemented as three-layer (256 → 64 → 30 units) and four-layer (1000 → 200 → 100 → 30 units) feed-forward networks, respectively. All layers used linear transformations with batch normalization and ReLU activation, except for the final layer. Encoders mapped per-cell inputs into 30-dimensional embeddings, which were then averaged across all cells belonging to the same patient to obtain patient-level, fixed-length, modality-specific vectors (Fig. 7a). The resulting patient-level embeddings were normalized using layer normalization [34] and concatenated. For multimodal integration, a gating fusion layer, a 60 → 60 linear layer followed by sigmoid activation [35] reweighted the concatenated [FC ‖ RNA] representation before being passed to the shared prediction head—a multilayer perceptron with hidden layers of 64, 32, and 16 units, each followed by batch normalization and ReLU activation, and an output layer producing a single logit value. Training was performed in batches of 1000 cells drawn from 10 patients at a time. Within each patient, up to 1000 cells per modality were randomly subsampled/batch to reduce computational cost and sampling variance, whereas validation used all available cells for each patient. The model was optimized using Adam [35] (learning rate 1 × 10^−3^, weight decay 1 × 10^−3^) for up to 200 epochs with a cosine-annealing learning-rate schedule [36]. Training minimized the binary cross-entropy loss with logits, with class weights inversely proportional to class frequencies applied to account for class imbalance (e.g. HD vs. pan-cancer). Model performance was evaluated using leave-one-out cross-validation at patient level, ensuring that both training and prediction were performed strictly at the patient level. Single-modality variants of the deep-learning model (sc-FC-only or sc-RNA-seq-only) used the same encoder and prediction head architectures and were trained with the same optimization and evaluation settings, but omitted the concatenation and gating fusion step. This enables direct comparison between single-modality and multimodal models, serving as an ablation-style assessment of the contribution of each modality. To interpret model predictions, we used Integrated Gradients (IG) [37], computing per-cell, per-feature attribution scores for each modality with 50 interpolation steps. The IG baseline was defined as mean feature vector of the corresponding cell-type population in the training set; for cells with ambiguous or multiple gate-based annotations, baseline was taken as average mean vectors across associated cell types. To (i) benchmark the multimodal deep-learning model, (ii) evaluate performance relative to models using clinical data alone, and (iii) examine which cell types were most informative for cancer prediction in sc-RNA-seq, we constructed a series of logistic regression models [35] across multiple data modalities. The regression analysis included four data types: (i) pseudo-bulk immune cell frequency matrices derived from sc-FC, (ii) pseudo-bulk gene expression matrices derived from sc-RNA-seq, (iii) serum cytokine profiles, and (iv) clinical variables. For pseudo-bulk gene expression, we used the same set of highly variable genes and the cell-type annotations defined in the sc-RNA-seq data analysis and QC workflow to construct both cell type–specific and global pseudo-bulk expression matrices. Features missing in >30% of patients were excluded; in the clinical dataset, this primarily affected complete blood count differentials. Data from 77 patients were available, of which 66 had complete coverage across all modalities and were used for model training. In total, 25 modality combinations were evaluated: 15 combinations of global data modalities (e.g. clinical + cytokines) and 10 models based on cell type–specific pseudo-bulk RNA-seq matrices, from which the top-performing models were selected for comparison with the multimodal deep-learning model (Fig. 7c). Each model was assessed in two binary classification settings: (i) one disease versus each of the other four diseases (10 pairwise comparisons, e.g. HCC vs. CRC), and (ii) one disease versus all other cancers combined as a pan-cancer group (5 comparisons, e.g. HCC vs. pan-cancer), resulting in a total of 375 trained models using a standardized pipeline comprising median imputation, *z*-score normalization, and L1-penalized logistic regression (liblinear solver, balanced class weights to account for class imbalance, maximum 10 000 iterations). Model performance was evaluated by receiver operating characteristic area under the curve [38] (ROC–AUC) under leave-one-out cross validation (see Supplemental Fig. 5 for detailed cross-validation results for all trained models). To assess whether classification performance could be explained primarily by demographic covariates rather than cancer-associated features, we additionally trained baseline models using only age and sex as input variables. These covariate-only baselines were implemented using the same preprocessing and leave-one-out cross-validation framework as the other linear models. To further account for potential nonlinear effects of covariates, we additionally trained Random Forest classifiers using age and sex only (n_estimators = 500). The performance of these covariate-only models is summarized in Supplemental Fig. 5. To visualize key pathways in each gene cluster, we defined gene–gene distance as 1 − Pearson correlation of expression profiles and applied Leiden clustering [39] to identify correlated gene modules. For each module, top 5% of genes with the highest IG attribution scores were selected for pathway enrichment analysis. Finally, genes were embedded into a two-dimensional space using t-distributed stochastic neighbour embedding (t-SNE) based on these distances, and clusters along with their corresponding enriched pathways were visualized (Fig. 8a).

### Statistical information

All values are presented as mean ± SD. Statistical evaluation was performed using GraphPad PRISM software, version 10.6.0 (GraphPad Software, San Diego, CA, USA; RRID:SCR_002798). The criterion for significance was set at *P* <.05. After proving the assumption of normality (Shapiro–Wilk test), one-way ANOVA or *t-*test was performed. Tukey’s multiple comparisons test was applied to control for type I error. This method controls the family-wise error, ensuring that the probability of false positives across the tested parameters is minimized. If the normality test failed, the Kruskal–Wallis (Dunnett’s multiple comparisons test) or Mann–Whitney *U* test was performed. The following symbols were used: ^*^ versus healthy donor; ^#^ versus tumour (entity specific).

## Results

### Patient characteristics and general clinical data

The study cohort comprised 66 cancer patients and 11 healthy donors (HD, Fig. 1a, which provides an overview of the experimental and computational workflow; detailed patient characteristics are listed in Supplemental Tables 1–4). Key demographic and clinico-pathological parameters are summarized in Table 1. Patients were diagnosed with one of the following solid tumours: glioblastoma (GBM, *n* = 11), colorectal cancer (CRC, *n* = 20), hepatocellular carcinoma (HCC, *n* = 16), or head and neck squamous cell carcinoma (HNSCC, *n* = 19). The median age was 65.3 years (range 33.5–85.6), with 31.8% female and 68.2% male. Also, 28.8% of the patients reported tobacco or alcohol use. Sixteen patients (24.2%) had a prior history of cancer, including 12 with documented malignancies and 4 with benign neoplasms, consistent with second malignancy rates observed in cancer registries [40]. One-third of the cohort was classified as obese (mainly grade I), with a higher proportion in the CRC group. Obesity is a known risk factor for secondary primary cancers [41] and in our small cohort, two-thirds of the obese patients were cancer survivors (1× prostate cancer, 1× breast cancer). Cardiovascular risk factors, including arterial hypertension and diabetes, were common, and several patients had established cardiovascular disease.

### Peripheral blood parameters and plasma cytokines reveal systemic immune dysregulation in cancer

General blood-based serum and haematological markers were either mildly elevated (e.g. C-reactive protein, CRP) or remained within normal range (Fig. 1b, c). Stratification by cancer type revealed mildly elevated CRP in all patients except GBM, which instead exhibited increased leukocyte counts (*P* < .05 and *P <* .01 vs. CRC and HCC, respectively), consistent with a neutrophil-driven inflammatory profile (Fig. 1c) [42]. Platelets were normal except in HCC with reduced counts, likely reflecting underlying liver pathology (Fig. 1c) [43]. Liver function declined with advancing tumour stage, as reflected by a strong correlation with the Child–Pugh score (Pearson’s *r* = 0.79, *P* < .001; Fig. 1d, Supplemental Table 1) and elevated liver enzymes (Fig. 1e, Supplemental Table 1).

**Figure 2:**
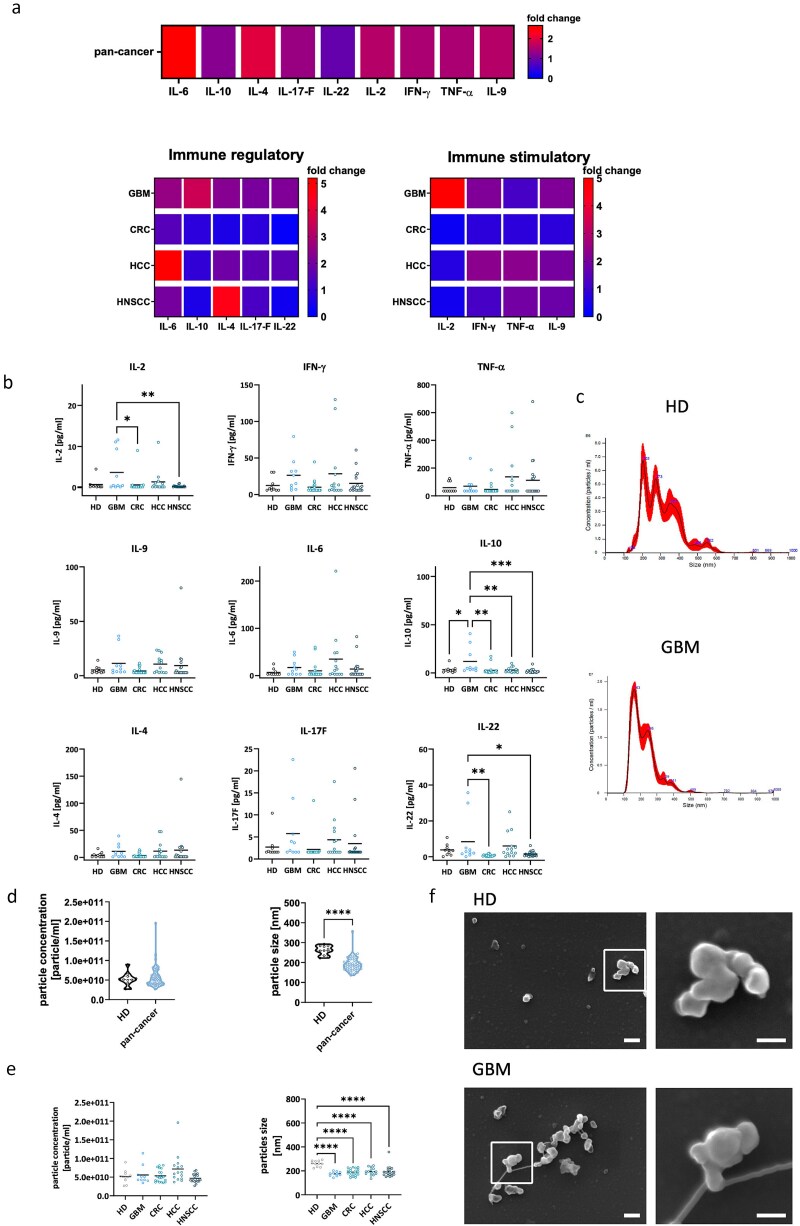
Cytokine profiles in plasma samples and amount of plasma EVs. (a) Heatmap showing the mean value of the fold change of each cytokine in the respective patient group compared to HD. (b) Quantitative analysis of cytokines. Data are shown as scatter plot + median. Kruskal–Wallis (Dunn’s multiple comparisons test), #*P* < .05, ##*P* < .01. HD, healthy donor; GBM, glioblastoma; CRC, colorectal cancer; HCC, hepatocellular cancer; HNSCC, head and neck squamous cell carcinoma; HD: *n* = 10; GBM: *n* = 10; CRC: *n* = 20; HCC: *n* = 14; HNSCC: *n* = 19. (c–f) isolation and characterization of plasma EVs. (c) Representative plots of nano particle tracking analysis (NTA) showing the size distribution of EVs (d–e). Size (nm) and concentration (particle/ml) of EVs were obtained by NTA measurement. Data are shown as violin plot + median and individual dots, unpaired *t* test, ^****^P < .0001. HD: *n* = 11; pan-cancer *n* = 64. (e) Data are shown as scatter plot + median. Kruskal–Wallis (Dunn’s multiple comparisons test), #*P* < .05, ##*P* < .01. (f) Scanning electron microscopy. Representative images shown for EVs from a HD and a GBM sample. Note the EV aggregation in the GBM sample, likely reflecting individuum-specific EV characteristics. Scale bars are 200 nm and 100 nm (inserts).

Multiplex cytokine analyses discovered elevated plasma IL-6 and IL-4 levels and reduced IL-22 levels across patients (2.6-fold and 2.0-fold vs. HD, and 0.89-fold vs. HD, respectively), though with considerable inter-patient variability (Fig. 2a). Among cancer types, GBM patients had the highest plasma levels of IL-2, IL-10, and IL-22 (Fig. 2b), underscoring disease-specific differences superimposed on a shared background of immune perturbation.

Total plasma EV concentrations were not significantly higher in cancer patients compared to HD; however, EVs from cancer patients were significantly smaller in both modal and mean size (*P <* .0001 vs. HD; Fig. 2c–e), regardless of tumour type. Scanning electron microscopy further revealed structural differences, including distinct EV shapes and sizes in individual cancer patients and HDs (Fig. 2f). As EV data were not incorporated into the multimodal modelling framework, these analyses are presented as complementary observations of systemic alterations. Together, these data indicate that cancer is associated with detectable, yet heterogeneous, systemic immune and vesicular alterations in peripheral blood.

### Distinct immune landscapes indicate peripheral immune dysregulation across cancer patients

**Figure 3:**
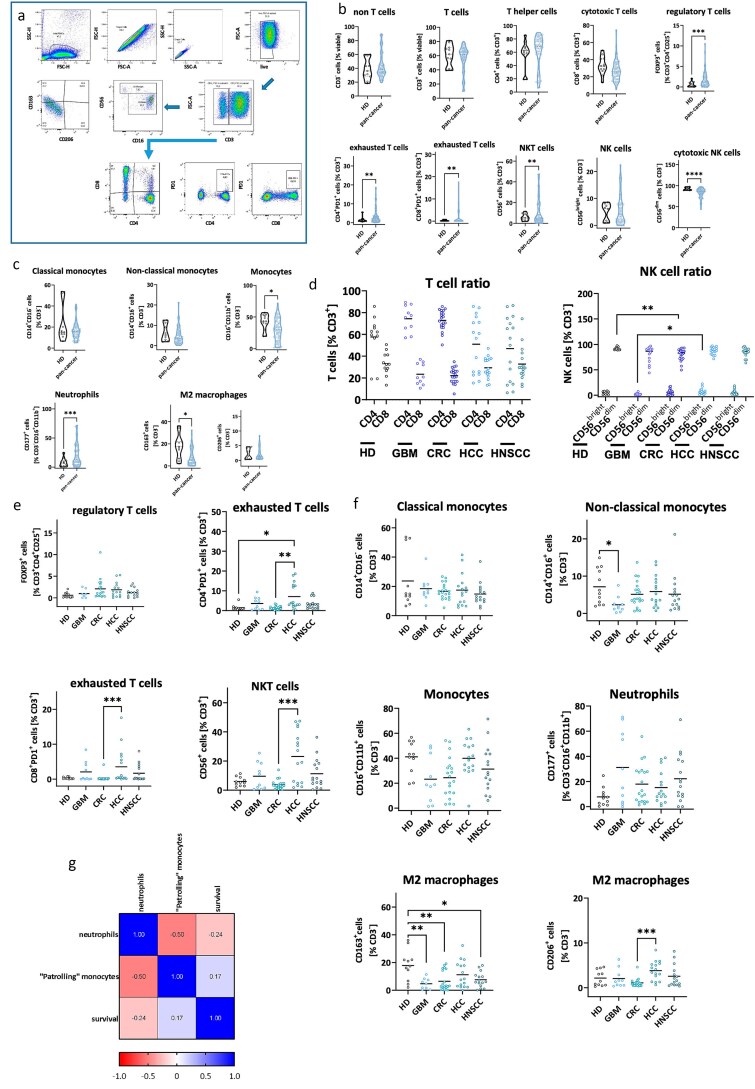
Spectral flow cytometry of whole blood samples. Fresh frozen blood was stained as described in the “Material and methods” section. A total of 50,000 events were measured in a live gate. (a) Gating scheme. (b–f) Quantitative analysis of immune cell phenotyping. (b, c) Quantification of the pan-cancer cohort. Data are shown as violin plots with individual dots + median, unpaired *t* test with Welch’s correction, ^*^P < .05, ^**^P < .01, ^***^P < .001, ^****^P < .0001. HD *n* = 12, pan-cancer *n* = 62. (d–f) Quantification stratified by cancer type depicted as scatter plot + median. Kruskal–Wallis test (Dunn’s multiple comparisons test), ^*^P < .05, ^**^P < .01, ^***^P < .001. HD, healthy donor; GBM, glioblastoma; CRC, colorectal cancer; HCC, hepatocellular cancer; HNSCC, head and neck squamous cell carcinoma; HD: *n* = 11; GBM: *n* = 10; CRC: *n* = 20; HCC: *n* = 16; HNSCC: *n* = 16. (g) Correlation matrix showing the association between myeloid-subtypes and survival of GBM patients. Patrolling monocytes are defined by the surface expression of CD11b^+^ and CD16^+^ and neutrophils by the expression of CD11b^+^, CD16^+^, and CD177^+^ Pearson *r* analysis.

Spectral immunophenotyping by sc-FC revealed widespread alterations in peripheral immune cell composition (Fig. 3). Cancer patients tended to have a lower proportion of CD8^+^ cytotoxic T cells (*P* = .08, Fig. 3b) and an increase in FOXP3^+^ regulatory T cells (*P <* .001, Fig. 3b). Elevated PD1 expression on both CD4^+^ and CD8^+^ T cells further indicated systemic T-cell exhaustion (*P* < .01, Fig. 3b). While NKT cell numbers were also elevated, the frequency of CD56^dim^ NK cells, the primary cytotoxic subset of NK cells [44], was significantly reduced (*P* < .0001, Fig. 3b).

We also observed marked changes in myeloid populations. There was a reduction in CD16^+^CD11b^+^ monocytes (*P* < .05, Fig. 3c). In contrast, CD177^+^ neutrophils were significantly enriched (*P* < .001, Fig. 3c). Among additional monocyte subsets, CD163^+^ cells were significantly reduced, while CD206^+^ monocytes remained unchanged—findings that align with altered myeloid phenotypes frequently observed in cancer [45, 46].

Stratified analysis across cancer types revealed disease-associated immune patterns superimposed on a shared background of immune dysregulation (Fig. 3d–g). CD4^+^:CD8^+^ T ratios were not significantly altered between patient groups, whereas CD56^dim^ NK cells predominantly decreased in CRC patients (*P* < .01 vs. HD Fig. 3d). T-cell exhaustion was most pronounced in HCC patients, in which the number of NKT cells was additionally higher than in the other cancer patients (*P* < .001 vs. CRC, Fig. 3e). Non-classical monocytes were also notably reduced in GBM patients (*P* < .05 vs. HD, Fig. 3f), counterbalanced by higher proportions of CD177^+^ neutrophils, although two clusters were evident across individual patients (i.e. high vs. low, Fig. 3f). Further stratification revealed a weak negative correlation between CD177^+^ neutrophils and patient survival (Pearson *r* = −0.24, Fig. 3g). Higher levels of ‘patrolling’ monocytes were only marginally associated with better outcomes (Pearson *r* = 0.17, Fig. 3g). Significantly lower levels of CD163^+^ macrophages were observed in GBM, CRC, and HNSCC patients, reflecting extensive immune dysregulation, while in HCC patients, proportions of CD163^+^ macrophages were not significantly altered (Fig. 3f).

Deeper profiling of the cancer-history cohort revealed no significant differences compared to those without prior malignancy (Supplemental Fig. 1). A tendency toward reduced T-cell numbers, counter-regulated by higher numbers of myeloid cells (e.g. CD177^+^ neutrophils), was evident, but this did not reach statistical significance, primarily due to high inter-patient variability (Supplemental Fig. 1).

### Sc-RNA-seq analysis reveals high variations in the gene signatures of monocytes across cancer patients

**Figure 4:**
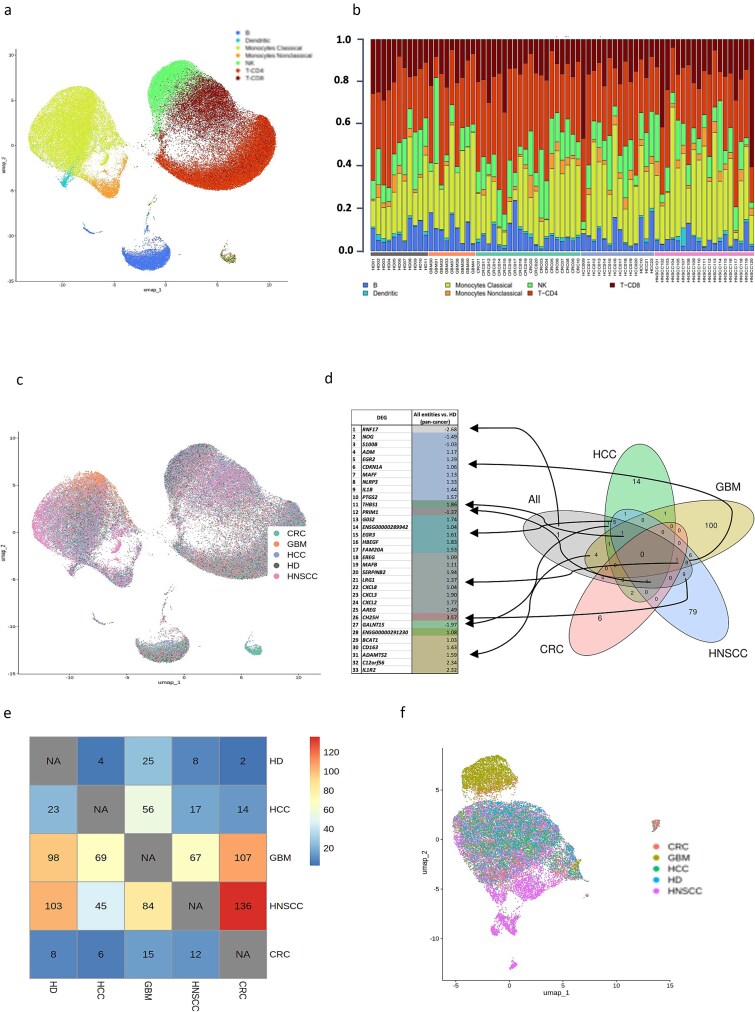
Technical setup for the sc-RNA-seq approach. (a) UMAP by cell type. (b) Relative cell count by cell type for each patient. (c) UMAP of all cells by patient group and healthy donor. (d) Venn diagram of differentially expressed genes compared to healthy donors. (e) Heatmap of differentially expressed genes in all cells (row: high expression; column: low expression). (f) UMAP of classical monocytes by patient groups and healthy donors.

A total of 100 373 cells from 73 peripheral blood samples—including a spike control for each sequencing run—were profiled across seven independent sc-RNA-seq runs. There were no batch effects between runs. Immune cell identities were annotated using *celltype* classifier, identifying seven major immune cell types: B cells, dendritic cells, classical monocytes, non-classical monocytes, NK cells, CD4^+^, and CD8^+^ T cells. Among these, CD4^+^ T cells and classical monocytes were the most abundant (Fig. 4a, b). Dimensionality reduction and clustering analyses revealed limited segregation by disease group, except for GBM and, to a lesser extent, CRC (Fig. 4c).

**Figure 5:**
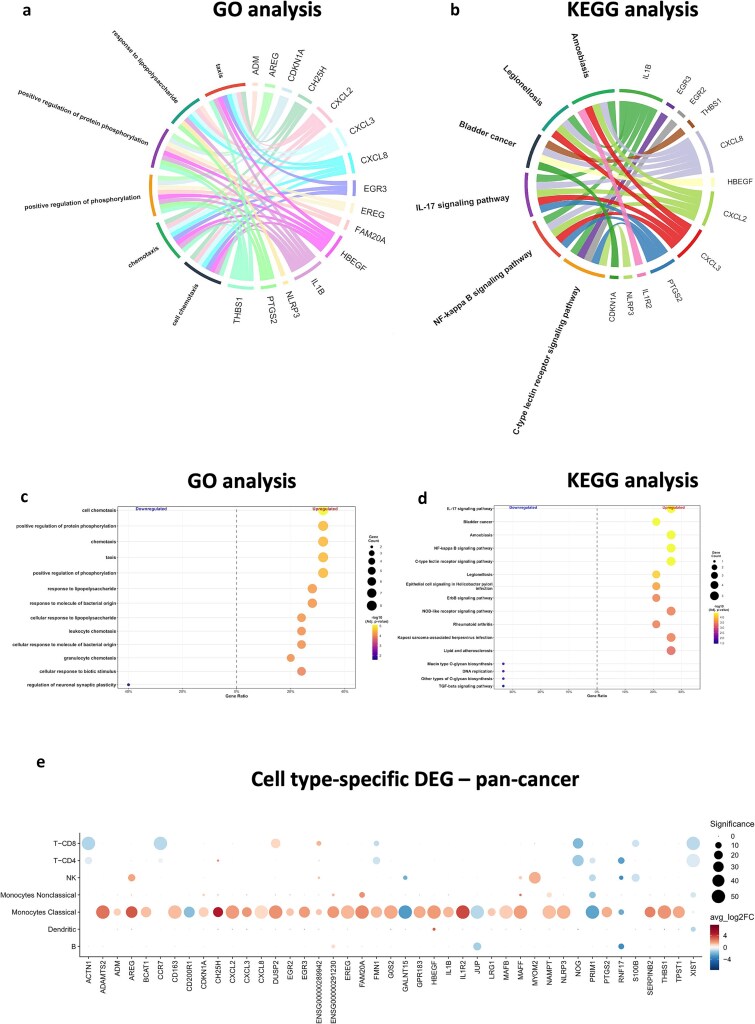
sc-RNA-seq approach. (a, b) GO and KEGG pathway enrichment analysis of differentially expressed genes in immune cells from cancer patients. Chord plot showing GO and KEGG biological processes enriched in differentially expressed genes (DEGs) across major immune cell types in cancer patients compared with HDs. Each ribbon connects a DEG (either up- or downregulated) to its associated (a) GO or (b) KEGG term, illustrating shared or gene-specific pathway involvement. (c, d) Functional enrichment of up- and downregulated genes in immune cells from cancer patients. Bubble plot illustrating enriched (c) GO terms and (d) KEGG pathways based on upregulated (yellow/orange) and downregulated (blue/violet) genes across major immune cell types. Bubble size represents the number of differentially expressed genes per pathway, while colour denotes regulation direction. Enrichment significance is indicated by –log2 (FDR-adjusted *P*-value). Key enriched pathways include chemokine signalling. Enrichment analysis was performed using [gProfiler, cytoscape], with FDR <0.05 considered significant. (e) DEGs across immune cell subsets in cancer patients. Bubble plot displaying the number and regulation direction of DEGs across immune cell types. Each bubble represents the DEG count within a given cell type. Bubble size corresponds to the number of DEGs, and colour indicates the direction of regulation—red for upregulated and blue for downregulated genes (cancer versus HD). Notable enrichment was observed in classical monocytes. DEGs were defined using |log₂ fold change| > 1 and FDR <0.05.

Differential gene expression (DEG) analysis identified 32 genes significantly dysregulated in cancer patients compared to HDs, independent of cancer type. Notably, no single gene exhibited uniform dysregulation across all cancer types (Fig. 4d). Cancer type–specific analysis identified the highest DEG burden in GBM and HNSCC patients (98 up- and 25 downregulated genes in GBM patients, 103 up- and 8 downregulated genes in HNSCC patients, Fig. 4e), whereas PBMCs from patients with gastrointestinal tumours showed fewer DEGs (23 + 4 DEGs in CRC patients and 8 + 2 DEGs in HCC, Fig. 4e, Supplemental Fig. 3a, d). Consistent with this, UMAP analyses show GBM and HNSCC-specific clustering in classical monocytes (Fig. 4f). Among the most consistently downregulated genes across all cell populations were *RNF17, GALNT15, NOG, PRIM1*, and *S100B*. Conversely, *CH25H, IL1R2, SERPINB2, CXCL3*, and *THBS1* were among the most strongly upregulated genes in pan-cancer patients relative to HD (Fig. 4d, Supplemental Fig. 2a). In addition to the aforementioned genes, *MAOA* and *ALOX15B* were among DEGs most highly upregulated in immune cells of GBM patients (Supplemental Fig. 2b). Across all cell types, genes related to chemotaxis (GO analysis; Fig. 5a, c) and immune modulation (KEGG analysis; Fig. 5b, d) were enriched. Further pathway-level interrogation confirmed upregulation of tumour-promoting processes, including chemotaxis and proinflammatory signalling (Nf-κB, ErbB). Cell type–specific analysis revealed that the majority of transcriptional changes occurred in classical monocytes (Fig. 5e, Supplemental Fig. 4b), with enrichment of pathways involved in chemotaxis and myeloid cell migration (Supplemental Fig. 2c–d).

**Figure 6:**
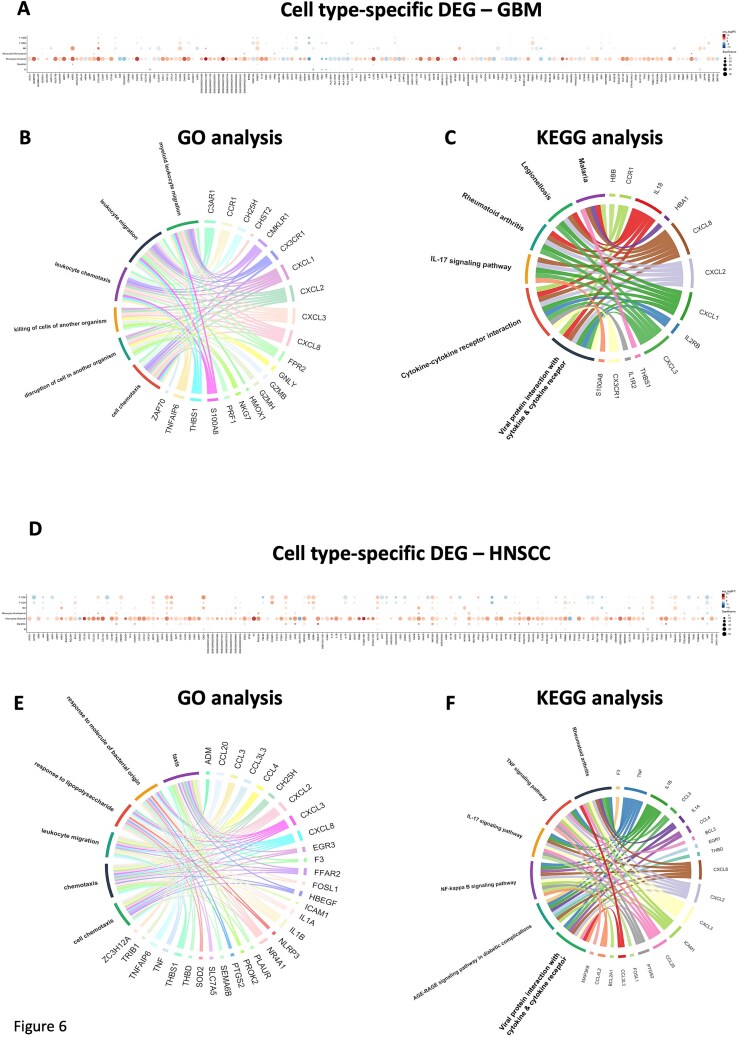
sc-RNA-seq approach. (a) DEGs across immune cell subsets in GBM patients. Bubble plot displaying the number and regulation direction of differently expressed genes (DEGs) across immune cell types. Each bubble represents the DEG count within a given cell type. Bubble size corresponds to the number of DEGs, and colour indicates the direction of regulation—red for upregulated and blue for downregulated genes (cancer vs. HD). Notable enrichment was observed in classical monocytes. DEGs were defined using |log₂ fold change| > 1 and FDR < 0.05. (b, c) GO and KEGG pathway enrichment analysis of DEGs in immune cells from GBM patients. Chord plot showing GO and KEGG biological processes enriched in DEGs across major immune cell types in GBM patients compared with HDs. Each ribbon connects a DEG (either up- or downregulated) to its associated (b) GO or (c) KEGG term, illustrating shared or gene-specific pathway involvement. (d) DEGs across immune cell subsets in HNSCC patients. Bubble plot displaying the number and regulation direction of DEGs across immune cell types. Each bubble represents the DEG count within a given cell type. Bubble size corresponds to the number of DEGs, and colour indicates the direction of regulation—red for upregulated and blue for downregulated genes (cancer vs. HD). Notable enrichment was observed in classical monocytes. DEGs were defined using |log₂ fold change| >1 and FDR <0.05. (e, f) GO and KEGG pathway enrichment analysis of DEGs in immune cells from HNSCC patients. Chord plot showing GO and KEGG biological processes enriched in DEGs across major immune cell types in HNSCC patients compared with HDs. Each ribbon connects a DEG (either up- or downregulated) to its associated (e) GO or (f) KEGG term, illustrating shared or gene-specific pathway involvement.

Most DEGs were found in classical monocytes, particularly in GBM (*n* = 85) and HNSCC (*n* = 75) (Fig. 6a, d). Again, the dominant pathways involved were related to chemotaxis, cellular migration, and inflammation (Fig. 6b, c, e, f). In GBM monocytes, pathways related to MHC class II complex assembly and antigen presentation were significantly downregulated (Supplemental Fig. 2e, f).

### Predictive modelling framework for cancer and healthy state classification

**Figure 7:**
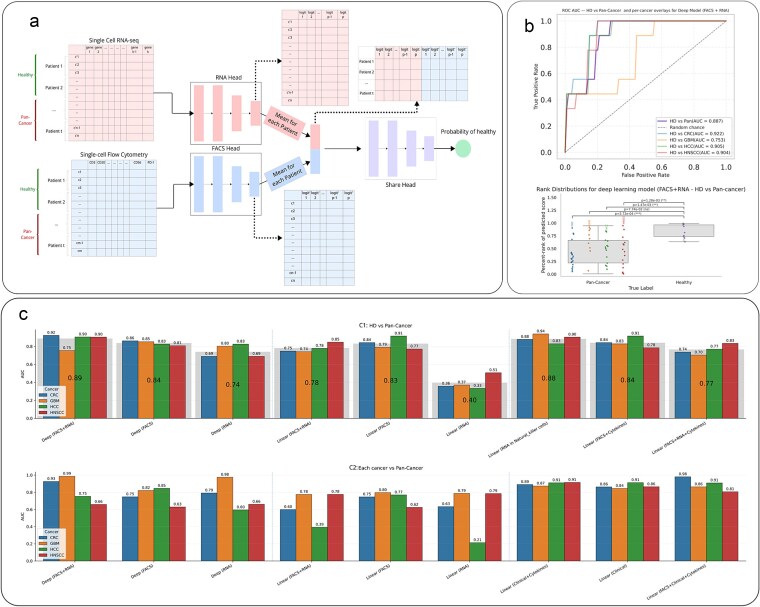
Overview of the multimodal deep-learning framework and its predictive performance. (a) Schematic architecture of the multimodal deep-learning model. The network receives cell-level features from both sc-RNA-seq and sc-FC data, which are encoded through separate modality-specific branches. Encoded features from all cells belonging to a given patient are averaged to generate patient-level embeddings. These embeddings, representing the same patients across modalities, are then fused and passed to a shared network for patient-level classification. (b) Performance of the multimodal deep-learning model in the HD versus pan-cancer classification task. The plot shows the receiver operating characteristic (ROC) curve of the model distinguishing healthy donors from pan-cancer. Additionally, ROC curves were computed separately for each cancer type within the pan-cancer group to assess performance across individual cancer subtypes. Examination of the model outputs revealed that HD patients received higher predicted scores compared to cancer patients, enabling clear separation between HD and pan-cancer groups. A Wilcoxon rank-sum test on the ranks of the predicted scores indicated significant HD–cancer differences for all subtypes except GBM. (c) Performance comparison of the multimodal deep-learning model with single-modality deep models and top linear models across HD versus pan-cancer and cancer type–specific settings. In the HD versus pan-cancer task, the multimodal framework outperformed all single-modality deep and linear models, achieving an AUC of 0.89. In cancer versus pan-cancer comparisons, it exceeded single-modality deep models for all cancers except HCC and outperformed corresponding linear models in all cases, as well as the top three linear models in CRC and GBM.

To investigate the ability to integrate multimodal single-cell data and explore predictive immune signatures within the collected sc-FC and sc-RNA-seq data, we set up a predictive multimodal deep-learning model (DL) for classification. This model integrates sc-RNA-seq and sc-FC data on a single cell level (Fig. 7a) to allow capturing multivariate, nonlinear relationships between low-level biological features and cancer types, and preventing information loss caused by manual preprocessing such as cell clustering and labelling. The model was trained to distinguish HD from pan-cancer patients (Fig. 7c.1) as well as distinguishing between cancer types (Fig. 7c.2), achieving robust performance across tasks. For this, a leave-one-patient-out cross-validation scheme was used, ensuring that predictions are only made for samples that have not been seen by the model during training. We compared DL to a variety of linear models based on individual or combined modalities, including clinical, cytokine, sc-RNA-seq, and sc-FC data. (For training linear models, sc-RNA-seq profiles were aggregated into patient-level pseudo-bulk expression and sc-FC features summarized as patient-level cell-type proportions.) Since the clinical data features themselves are well-known cancer-associated variables, such as elevated γ-GT levels in HCC, logistic regression based on clinical data performed well for cancer-type classification, as expected (Fig. 7c.2, Supplemental Fig. 5). DL performed consistently well across classification tasks, outperforming the clinical data–based logistic regression in CRC and GBM, and achieving its highest AUC in the HD versus pan-cancer (AUC = 0.89; Fig. 7b, c). Notably, this strong performance was achieved from raw sc-RNA-seq and sc-FC data, despite the small cohort of 77 patients with different cancer types, indicating that DL models can capture biologically informative patterns directly from single-cell multimodal measurements.

Comparison with single-modality DL models (sc-FC-only and scRNA-seq-only) showed that the multimodal model (sc-FC + scRNA-seq) achieved higher performance across most tasks (Fig. 7c). These comparisons constitute a controlled ablation study, with identical architecture and training while removing one modality at a time. This indicates that integration provides complementary predictive information beyond either modality alone. Integrated Gradients further confirmed that both modalities contribute to the model’s predictions (see Supplemental Fig. 6).

**Figure 8:**
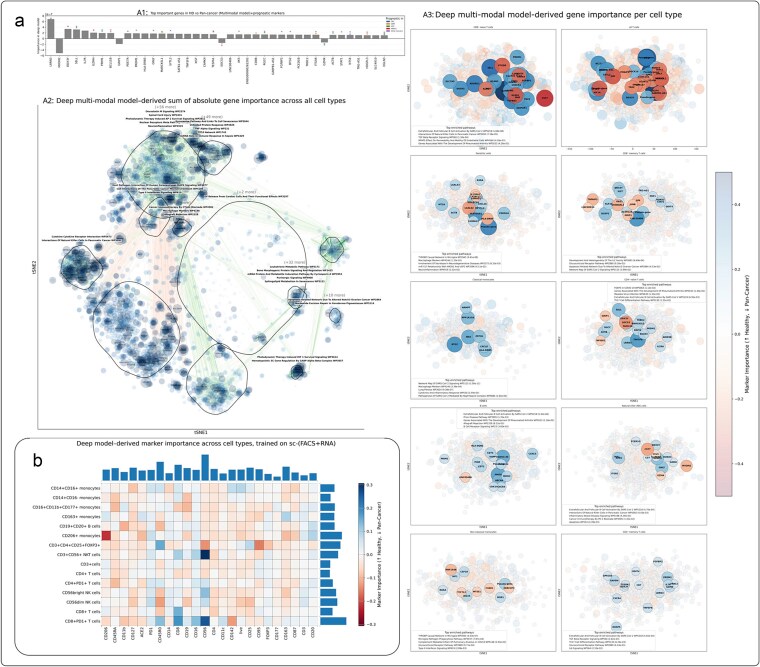
Biological patterns from the multimodal deep-learning model (HD versus pan-cancer). (a1) integrated gradients (IG) with a cell type–informed baseline was used to compute absolute gene importance scores, which were summed across all cells to estimate overall gene importance. Notably, 22 of the top 40 genes identified by the model were previously reported as prognostic in at least one cancer type (CRC, HNSCC, GBM, or HCC). (a2) Gene–gene distance was defined as *(1 − Pearson correlation)*, and Leiden clustering identified correlated gene modules. For each module, the top 5% most important genes (based on overall importance in the deep multimodal model) were selected for pathway analysis. The t-SNE plot shows gene–gene distances, with node size reflecting overall importance and annotated pathways indicating enriched biological processes per module. (a3) Cell type–specific visualization of gene importance derived from the deep multimodal model. Each panel represents one immune cell type, with genes positioned by pairwise similarity *(1 − Pearson correlation)*. Node size denotes overall gene importance, and highlighted pathways above clusters represent enriched biological processes among the top 5% most important genes. (b) Heatmap of averaged integrated gradients importance from the deep multimodal model applied to the sc-FC data used in the multimodal framework, shown per cell type (columns) and marker (rows). The top bar shows the sum of absolute importance across markers for each cell type, and the right bar shows the sum across cell types for each marker, highlighting the most informative cell types and FC markers.

To further investigate (i) whether DL captures plausible biological signals and (ii) whether it utilizes biological signals that may point towards candidate molecular features, we employed Integrated Gradients (IG), a method from the field of Explainable Artificial Intelligence, which can explain the model’s decisions in the HD versus pan-cancer task. Support for (i) comes from three converging lines of evidence. First, in the RNA branch, 22 of the top 40 IG-ranked genes have previously been reported as prognostic markers in CRC, HCC, HNSCC, or GBM, and 7 in other cancer types according to the Human Protein Atlas [47] (Fig. 8a.1). Second, pathway analysis of correlated top genes prioritized by the model’s IG importance scores revealed strong enrichment for immune and inflammatory pathways, including Type II Interferon (*P* = .000001), TNF-α signalling (*P* = .00006), and cytokine–cytokine receptor interaction (*P* = .003), highlighting immune activation as a defining feature to distinguish HDs from pan-cancer patients (Fig. 8a.2). Third, feature attribution in the FC branch was biologically concordant with known immunophenotypes. For example, CD206^+^ monocytes negatively influenced HD predictions, consistent with enrichment of M2-like tumour-associated macrophages in cancer. In contrast, elevated CD56 expression on PD1^+^ NKT cells was associated with predictions towards HD, reflecting cytotoxic immune phenotypes enriched in the latter (Fig. 8b). For (ii), the relevance of the identified pathways was further linked to specific cell types in cancer (Fig. 8a) such as the tumour suppressor *BTG2* in classical monocytes, *CST7* (Cystatin F) in NK cells, a known negative regulator of cytotoxicity, as well as *MTSS1*, involved in cytoskeletal regulation of non-classical monocytes.

**Figure 9:**
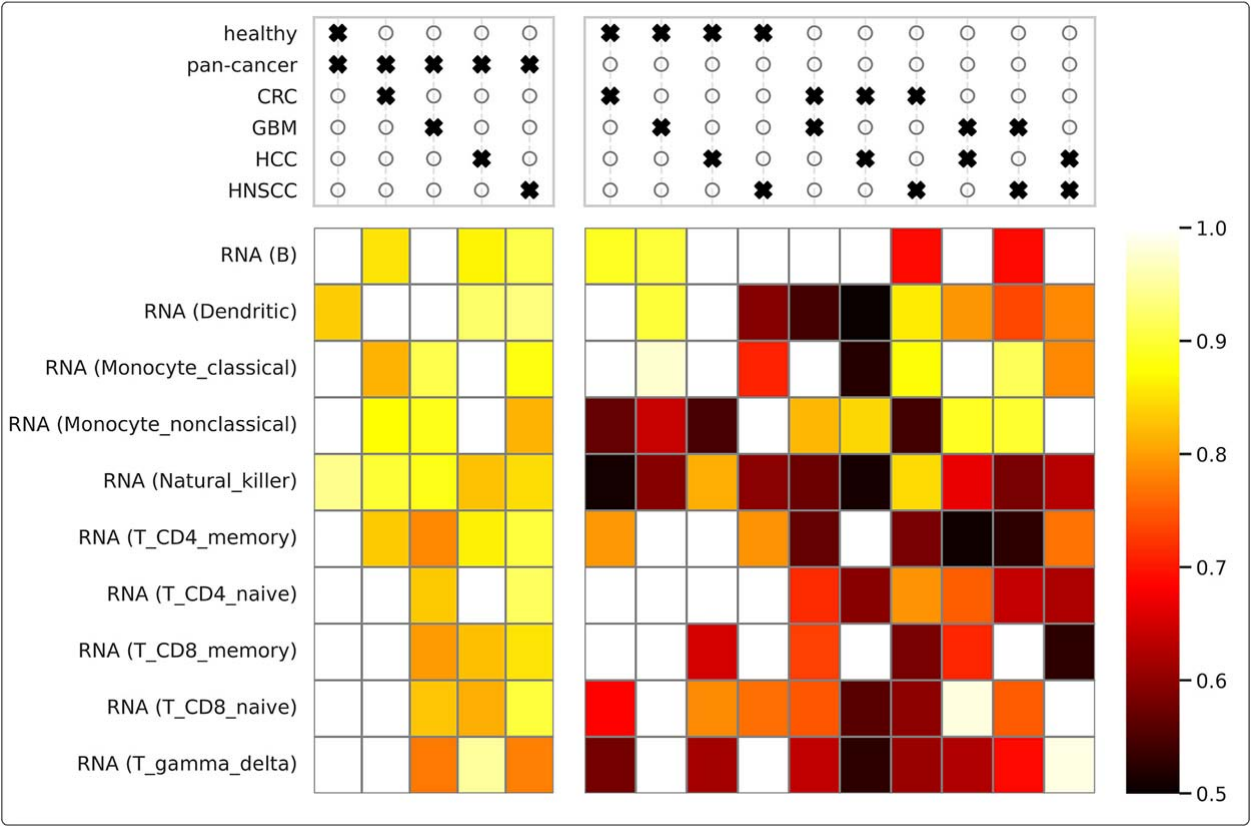
Performance of logistic regression models on pseudo-bulk gene expression profiles across cell-type populations in different binary classification settings. Each cell colour represents the AUC value of the binary logistic regression model for the classification task indicated at the top, based on pseudo-bulk gene expression profiles of the corresponding cell-type population. Only models with AUC >0.5 are shown in colour; cells corresponding to lower-performing models are displayed in white.

To further guide biological interpretation and inform future sampling and clinical modelling strategies, we quantified the predictive value of each cell type by training logistic regression models on pseudo-bulk RNA-seq profiles derived from sc-RNA-seq–defined populations. This analysis revealed which cell types carried the strongest predictive signals: in our dataset, NK and dendritic cells distinguished HD from pan-cancer; non-classical monocytes and NK cells were most predictive for CRC; monocytes and NK cells for GBM; dendritic and B cells for HCC; and B cells distinguished CRC from HD. Non-classical monocytes emerged as key discriminative populations across CRC versus GBM, CRC versus HCC, GBM versus HCC, and HNSCC versus GBM (Fig. 9).

## Discussion

Using a multi-omics framework, we integrated complementary single-cell and protein-level immune measurements to characterize cancer-associated systemic immune alterations and identify potential cancer-related biomarkers. Clinical laboratory data revealed subtle but consistent blood parameter changes in cancer, aligning with previous findings [48]. GBM patients showed elevated leukocyte counts, likely reflecting tumour-driven increases in mature neutrophils [42]. Platelet counts were generally normal across groups except in HCC, where marked thrombocytopenia was found. This likely reflected the combined influence of cirrhosis and active malignancy on thrombopoiesis and platelet turnover.

Plasma-based biomarkers, including leucocytosis, thrombocytopenia, and neutrophil-to-lymphocyte ratio (NLR), are established independent prognostic markers in cancer [49]. Our findings corroborate their clinical relevance, showing that systemic inflammatory markers mirror tumour–host immune interactions, offering accessible and cost-effective predictors of outcome, e.g. in patients receiving immune checkpoint inhibitor therapy [50]. For cancer types lacking validated biomarkers, advanced immunological techniques may improve diagnostic precision and therapeutic monitoring. Multi-colour flow cytometry, in particular, enables comprehensive immune profiling and real-time tracking of treatment-related immune shifts [51–53]. In our pan-cancer cohort, we identified pronounced immune dysregulation, T/NK cell imbalance, widespread T-cell exhaustion, elevated neutrophils, and altered myeloid phenotypes—consistent with tumour-driven phenotypic and functional immune changes [54]. Notably, these cellular alterations are concordant with the transcriptional features identified through integrative multimodal analysis, particularly within monocyte and NK cell compartments, supporting a coordinated, multilayered remodelling of peripheral immunity in cancer. The reduction in mature macrophages likely reflects systemic imbalance rather than simple depletion. While age- and gender-related immune variation may contribute to the observed differences, the cancer-type specificity and multimodal consistency of these alterations argue for a disease-associated component beyond immunosenescence [55, 56]. Consistent with this, covariate-only baseline models using age and sex achieved lower performance than models incorporating immune-derived features (Supplemental Fig. 5), although the results may still be partially influenced by these variables.

Immune cell alterations differed across cancer types. Notably, HNSCC patients exhibited only modest systemic immune changes despite having predominantly advanced disease. This finding suggests that blood-based markers alone may not adequately capture complexity of tumour-immune interactions. It highlights the importance of integrating peripheral immune profiling with tissue-derived biomarkers and patient-derived model systems to achieve more comprehensive biological insights and to improve clinical decision-making. In GBM and HCC patients, immune alterations were most pronounced, aligning with recent GBM studies reporting altered NK:T cell ratios across glioma subtypes and elevated B cell levels as markers of poor prognosis [57]. Although limited by sample size, elevated CD177^+^ neutrophil levels indicated poorer survival. In contrast, increased ‘patrolling’ monocytes—producers of proinflammatory cytokines (TNF-α, IL-12p40/p70) and cytotoxic reactive nitrogen intermediates [58]—correlated with more favourable outcomes. Nonetheless, their phenotypic and functional plasticity and their short lifespan constrain their utility as reliable and stable biomarkers [45, 59]. Given the limited sample size within individual cancer cohorts, cancer type–associated differences should therefore be interpreted as exploratory and hypothesis generating rather than definitive tumour-specific signatures.

Sc-RNAseq confirmed widespread immune dysregulation and uncovered distinct gene expression signatures across peripheral immune cell subsets. The most pronounced transcriptional changes occurred in classical monocytes, with enrichment in chemotaxis-related pathways. While no single gene was differentially expressed across all immune cell types and tumour entities, a small subset—*PRIM1, CH25H*, and *THBS1*—showed recurrent alterations across cancers and patients. These genes are established contributors to tumour progression [46, 60, 61], underscoring their potential as diagnostic and prognostic biomarkers [62]. *PRIM1* participates in DNA replication, damage repair, hepatocarcinogenesis, and potentially promotes neutrophil recruitment and CRC metastasis [63, 64]. *CH25H*, whose gene product is interferon stimulated [65, 66], catalyses the monooxygenation of cholesterol to produce 25-hydroxycholesterol (25HC). CH25H—and oxysterols more broadly—exhibit context-dependent, dichotomous roles in cancer. In pancreatic cancer and melanoma, CH25H suppresses tumour growth by depleting tumour cell cholesterol, inhibiting tumour EV uptake, and enhancing CD8^+^ T cell–mediated antitumor immunity [67, 68]. In melanoma, tumour lymphatic endothelial cell–derived 25HC also inhibits PPAR-γ in macrophages and monocytes, promoting their differentiation into proinflammatory iNOS^+^ myeloid cells within the tumour microenvironment [69]. Conversely, in CRC and other solid tumours, CH25H promotes epithelial-to-mesenchymal transition and tumour progression through tumour-associated myeloid-derived suppressor cells. These, in turn, suppress the cGAS–STING pathway, a key component of the innate immune system that detects cytosolic DNA and triggers inflammatory responses. Suppression of this pathway reduces the inhibitory activity of the downstream TBK1–RIPK3 complex on arginase 1, thereby accelerating immune escape [70]. Mechanistically, elevated lysosomal 25HC in macrophages competes with cholesterol for GPR155 binding, inhibiting mechanistic target of rapamycin complex 1 (mTORC1) activity. This triggers AMPKα activation, metabolic reprogramming, and phosphorylation of STAT6 at Ser564, collectively enhancing arginase 1 expression. Proinflammatory cytokines IL-4 and IL-13 can activate this pathway [65]. Notably, CH25H is detectable in clonally expanding CXCL13^+^CH25H^+^IL-21^+^PD-1^+^ CD4^+^ T helper cells following immune checkpoint blockade in HCC. The therapeutic response positively correlates with the abundance of these T-cell subsets within specialized intratumoral niches [71]. Moreover, circulating CH25H levels in pan-leukocytes may constitute a molecular marker for prognosis and risk stratification in lung cancer [62]. These findings elegantly highlight the dual and context-dependent roles of CH25H in cancer.

*THBS1* regulates cell–matrix interactions, angiogenesis, and immune modulation [72], and its elevated transcript levels in our pan-cancer cohort are consistent with previous reports linking *THBS1* to poor prognosis and brain metastasis in patients with HER2-enriched breast cancer [73], prostate cancer [74], and gastric cancer. The underlying molecular mechanism is aberrant methylation [75]. The functional domains of THBS1 interact with different receptors, activating multiple downstream signalling pathways. Through these interactions, THBS1 plays a fundamental role in regulating tumour behaviour. Notably, and similar to CH25H, it exhibits context-depending dual functionality, acting either as a tumour suppressor or a promoter, depending on its expression pattern and microenvironmental cues. In its suppressive role, THBS1 inhibits angiogenesis through interactions with integrin CD36 or TNF-α. Conversely, in a pro-tumorigenic context, it enhances invasiveness, proliferation, hypovascularity, and platinum resistance through TGFβ signalling and mesenchymal transition [76–78]. THBS1 also promotes the development of immunosuppressive myeloid-derived suppressor cells with low HLA expression through STAT3 and NF-κB signalling, thereby establishing an inflammatory regulatory network intertwined with oncogenic pathways [79]. Notably, THBS1 has recently been identified as a potential biomarker in EVs from prostate cancer patients [80]. Functionally, THBS1-containing EVs mediate communication between cancer cells and macrophages to aggravate M2 polarization via modulation of TGF-β1 [81]. Together, these features converge on pathways associated with myeloid activation, immune suppression, and inflammatory signalling, suggesting that peripheral immune alterations reflect systemic tumour-driven reprogramming of innate and adaptive immune compartments. These findings position *THBS1* as a putative pan-cancer immune-associated marker. Importantly, pharmacological targeting of THBS1 with gabapentin—an FDA-approved anticonvulsant—has demonstrated survival benefits in GBM [82]. Mechanistically, inhibition of THBS1 suppresses synaptogenesis and glutamatergic neuronal hyperexcitability, while alleviating macrophage-mediated T-cell suppression [83].

An unexpected finding was the different number of deregulated genes across cancer types, with GBM patients exhibiting a ten-fold higher DEG count than CRC patients. This likely reflects intrinsic biological differences and distinct clinical management strategies. Notably, 50% of CRC cases in our cohort were microsatellite instability-high (MSI-H), a subtype associated with pronounced immune activation and strong responsiveness to immunotherapy [84, 85]. However, we did not observe distinct immune-related transcriptional signatures between MSI-H and microsatellite-stable subtypes, suggesting that immune editing in CRC may be predominantly localized. Large-scale studies analysing peripheral immunity profiles are lacking.

Interestingly, a history of prior malignancy did not significantly affect immune parameters, indicating potential immune recovery between cancer episodes [86]—an insight relevant to survivorship and secondary malignancy surveillance. The need for minimally invasive tools is increasingly recognized. Recent evidence from the PREDICT trial demonstrated that time to treatment failure can be predicted from blood in pancreatic cancer, with an immune-active, treatment-naïve microenvironment correlating with improved second-line outcomes and a preserved immune signature post-chemotherapy [87]. These findings support the use of blood-based prognostic tools to guide future patient triage.

We developed a single-cell multi-omics deep-learning model that directly integrates raw sc-RNA-seq and sc-FC data, capturing both broad associations and fine-grained, nonlinear patterns. Importantly, the strength of this approach does not lie in identifying individual immune alterations, many of which are already known, but rather in their integrative, multimodal quantification and interpretation within a unified analytical framework. Since EV analyses were exploratory and not incorporated into the multimodal modelling framework, they should be interpreted as complementary observations. Despite the modest cohort size (77 samples), the model showed consistent within-cohort performance comparable to established clinical markers, highlighting the promise of DL approaches even in data-limited settings. Mechanistic examination showed that the model recovered known cancer-related biological processes, supporting its biological plausibility, while also identifying additional, previously unrecognized features that highlight the complexity of cancer-associated immune dysregulation. Although validating these specific processes is beyond the scope of this study, our findings provide proof-of-concept that raw single-cell multi-omics DL models can support biologically interpretable exploration of systemic immune alterations in cancer.

Several limitations should be acknowledged: (I) the single-centre, cross-sectional design without external validation may not account for regional variability; (II) blood was sampled at a single preoperative time point, precluding longitudinal assessment of immune dynamics; (III) the overall cohort size was relatively small, with limited representation of individual tumour types. Although class-weighted loss was used to mitigate this imbalance during training, the limited sample size in some classes may still affect the stability of sensitivity and specificity estimates. And (IV) while CRC was included, other prevalent cancers such as lung and breast cancer were not represented. In particular, the absence of an independent external validation cohort limits the assessment of model generalizability and necessitates cautious interpretation of classification performance derived from internal cross-validation. Although class-weighted loss was used to mitigate this imbalance during training, the relatively small healthy donor group may still affect the stability of sensitivity and specificity estimates. Given the complexity and plasticity of the peripheral immune landscape, future studies should include prospective, longitudinal sampling and external validation to better capture temporal immune dynamics.

In conclusion, our findings underscore the prognostic value of blood-based immune markers in oncology and highlight the importance of integrating systemic and tissue-derived biomarkers to develop robust frameworks for studying cancer-associated immune states and informing future diagnostic and monitoring strategies. While these features are not validated as clinical biomarkers, their consistent detection across modalities highlights their potential as candidates for future validation in independent cohorts and longitudinal studies.

**Key Points**

- Peripheral blood cells of treatment-naïve cancer patients exhibit distinct, perturbed immune states across tumour types.
- Integrating single-cell transcriptomic and protein-level immune data enables comprehensive characterization of systemic immune alterations.
- A machine learning–guided multimodal framework captures nonlinear relationships in heterogeneous immune data with biological interpretability.
- This approach provides a generalizable strategy for integrative analysis of multimodal immune profiling data in cancer.

## Supplementary Material

Berlin_suppl_figure_tables_bbag320

## Data Availability

Please address all correspondence and material requests to C.M. The single-cell RNA sequencing data generated in this study have been deposited in the Gene Expression Omnibus (GEO) https://www.ncbi.nlm.nih.gov/geo/query/acc.cgi?acc=GSE314004 (accession no. GSE314004) and are available for review using the token kdqnyssarzinbyp. Associated metadata are included in the repository.

## Code availability

The code used for data preprocessing, model training, cross-validation, explainability analysis, and figure generation in this study is available to reviewers via an anonymous repository: Code Link
