# Supplementary material for: Machine learning–guided multimodal profiling defines perturbed immune states at the time of cancer diagnosis
Source: Brief Bioinform. 2026 Jun 17;27(3):bbag320. doi: 10.1093/bib/bbag320 (PMC13274990; doi:10.1093/bib/bbag320)
Supplement: Berlin_suppl_figure_tables_bbag320 [file berlin_suppl_figure_tables_bbag320.pdf]

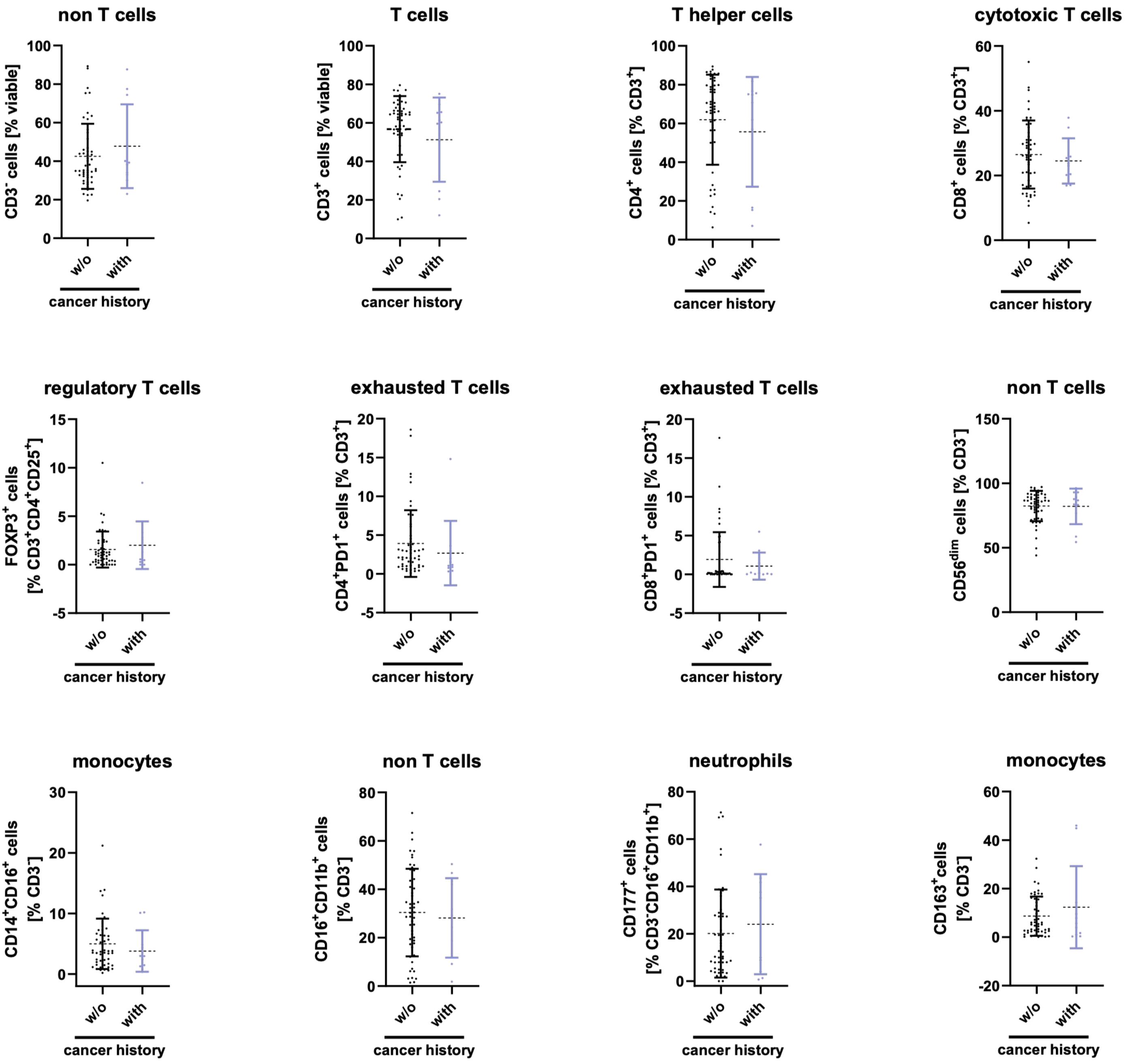

Supplemental Figure 1

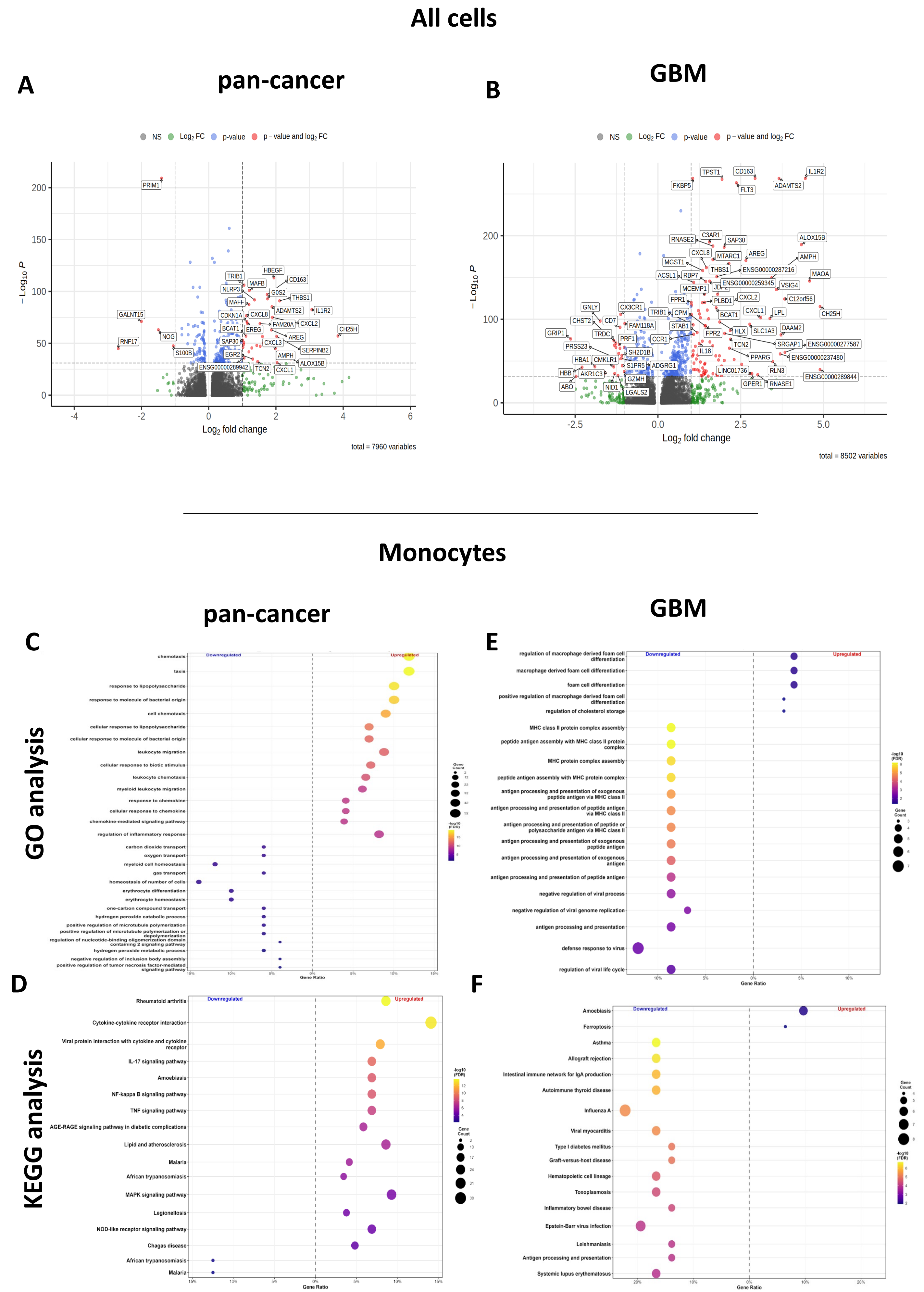

Supplemental Figure 2

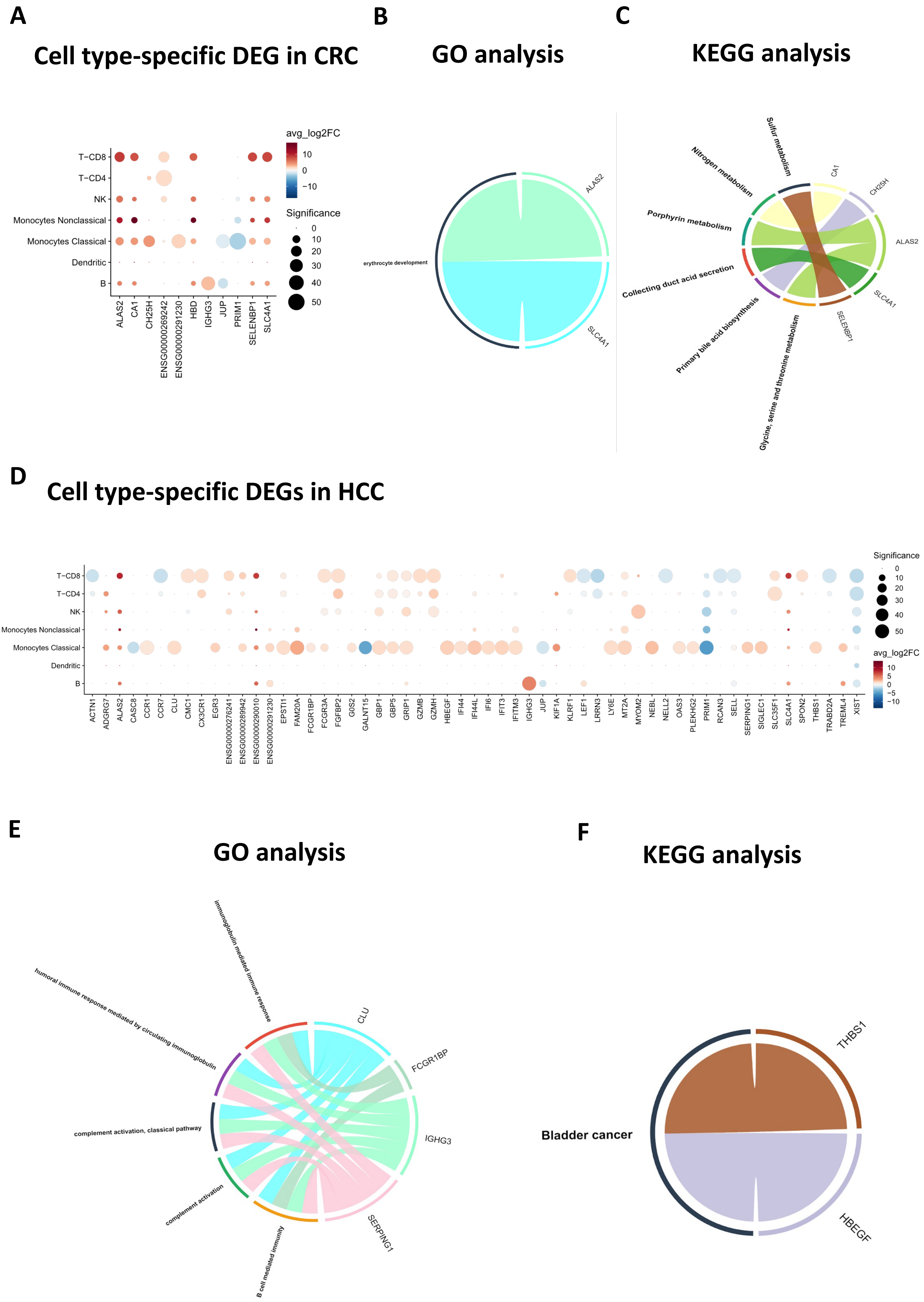

Supplemental Figure 3

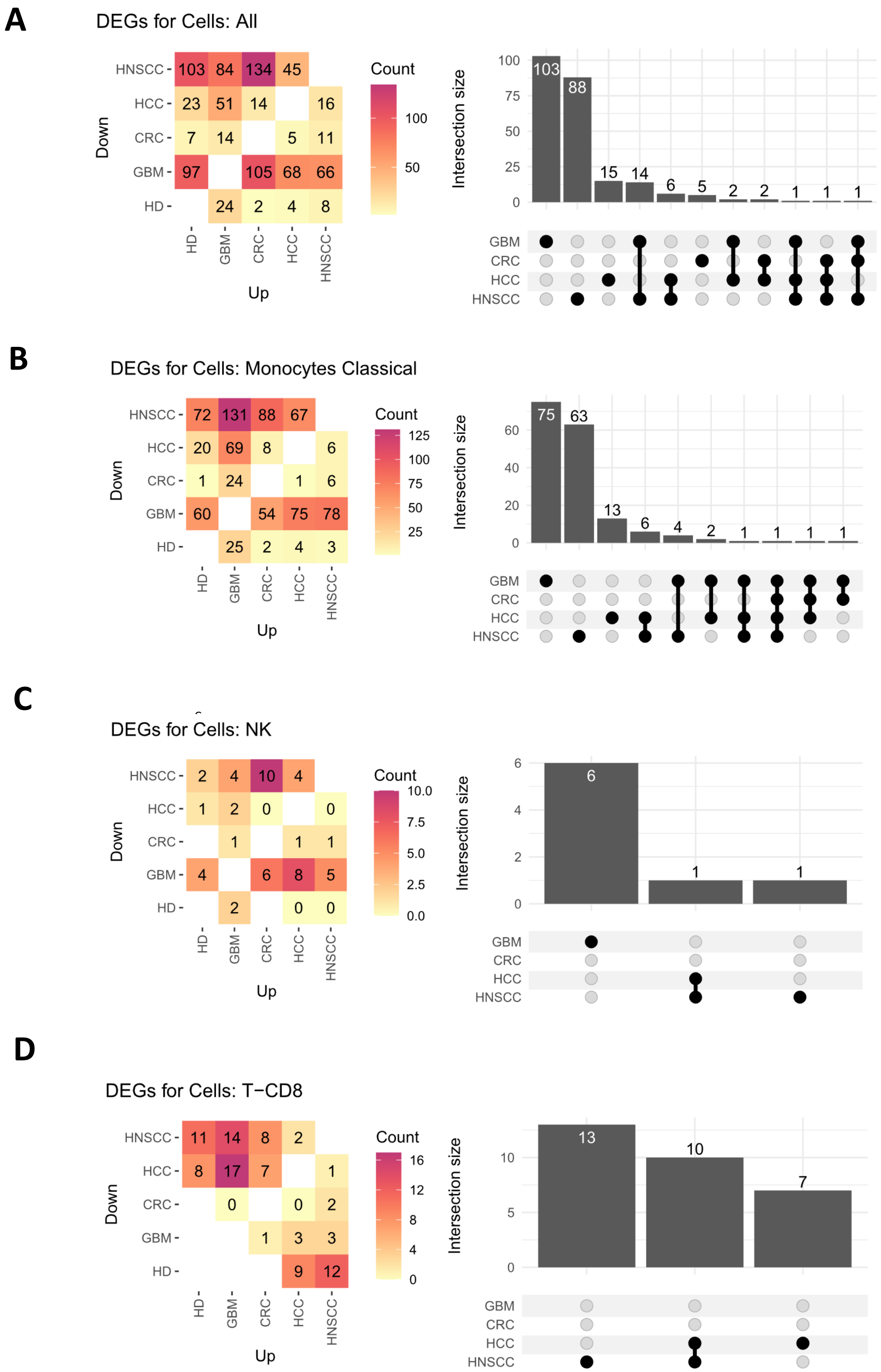

Supplemental Figure 4

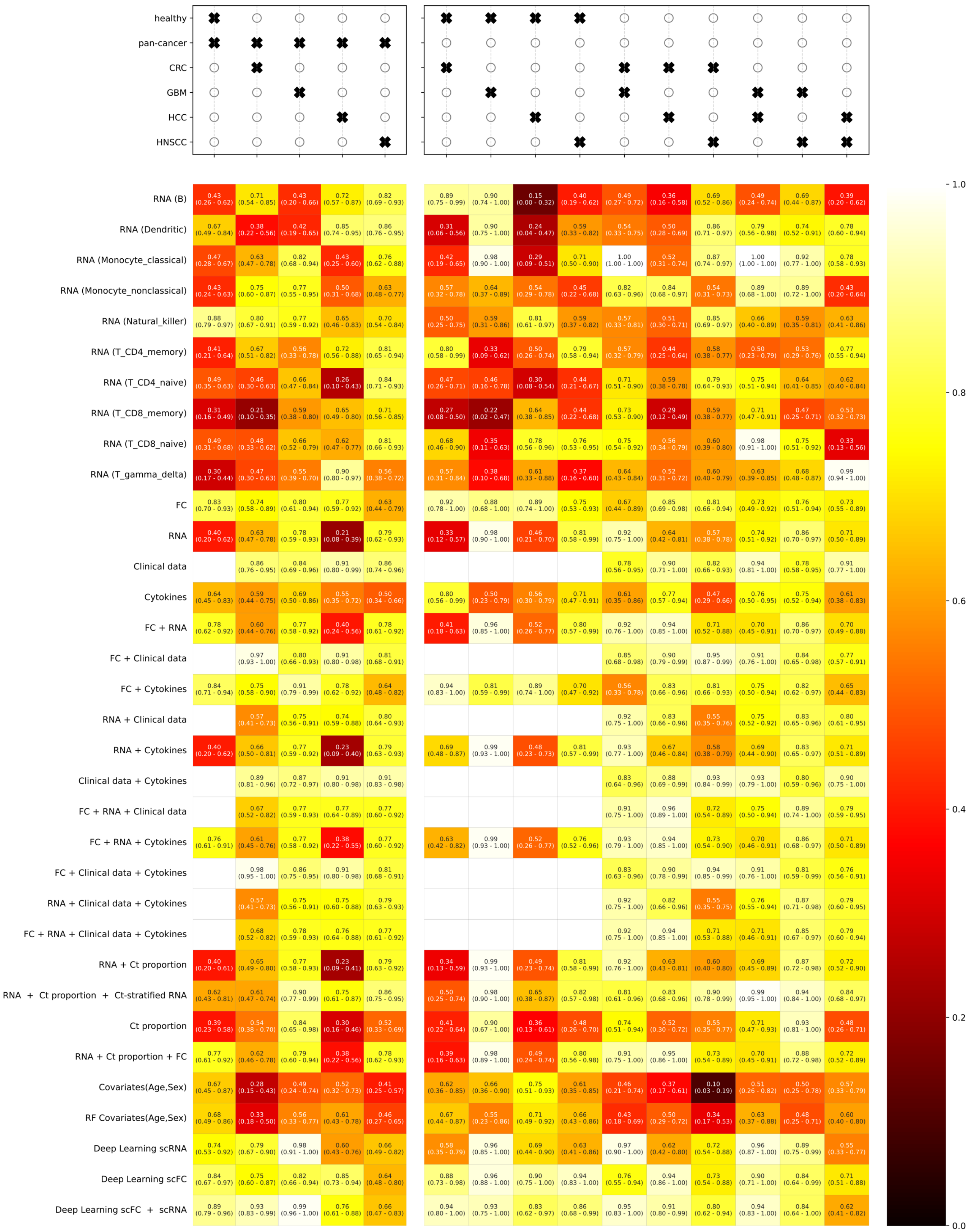

Supplemental Figure 5

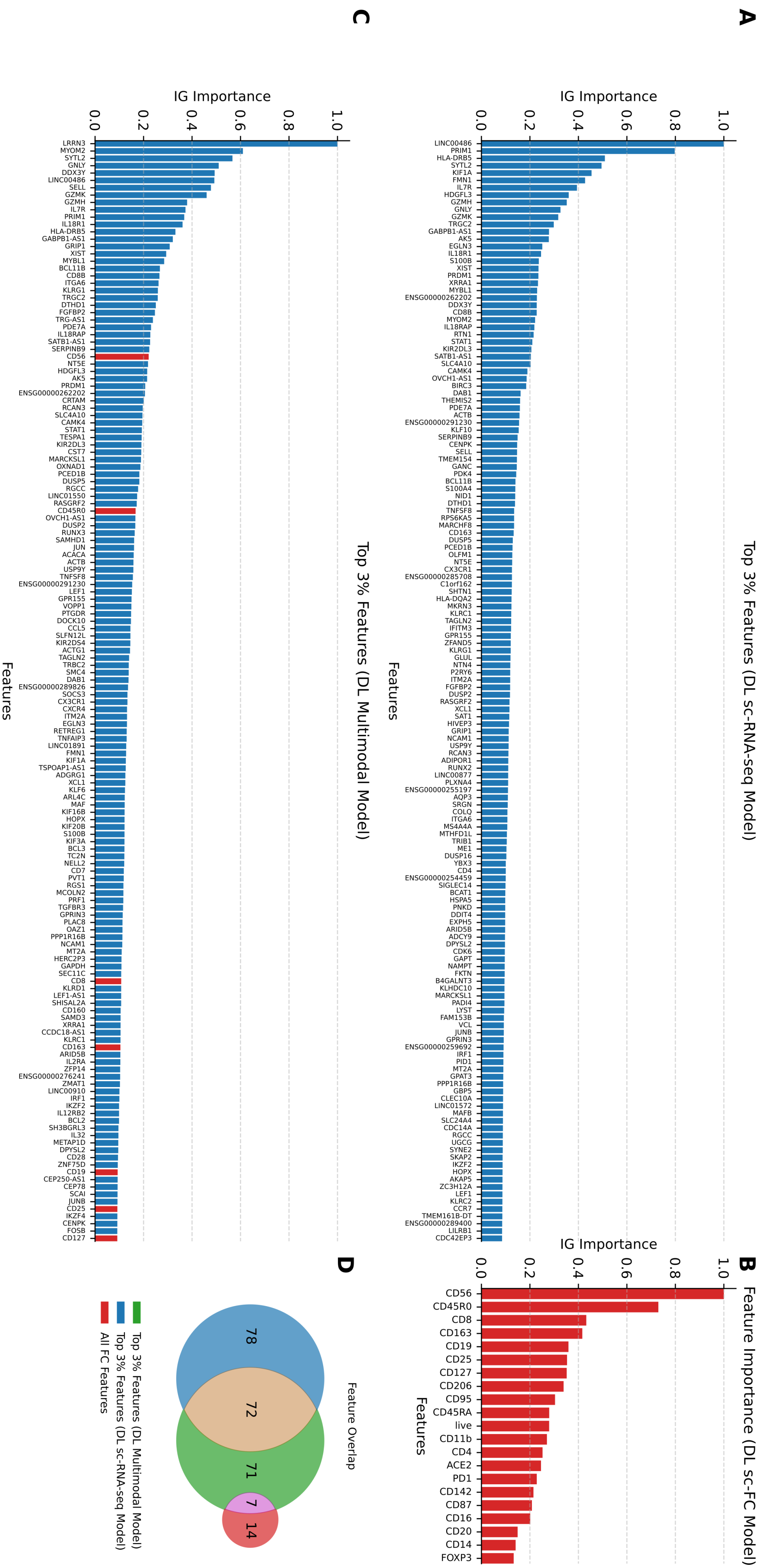

Supplemental Figure 6

Supplemental Table 1: Cancer staging and classification of liver function in the hepatocellular carcinoma patient cohort.

**Patient ID**	**BCLC stage**	**Distant metastases**	**Thrombosis**	**Child-Pugh score/stage**	**MELD score**	**Liver cirrhosis**	**Etiology**
**009**	A	no	no	5/A	9	yes	viral
**010**	D	no	suspected PVT	12/C	19	yes	unknown
**011**	B	no	no	6/A	8	yes	ethyltoxic
**012**	D	yes	PVT	10/C	17	yes	unknown
**013**	B	no	no	6/A	9	yes	unknown
**014**	C	yes	no	6/A	8	yes	unknown
**015**	A	no	no	6/A	8	yes	ethyltoxic
**016**	B	no	no	6/A	11	yes	unknown
**017**	A	no	no	6/A	7	yes	ethyltoxic
**018**	B	no	no	6/A	6	yes	viral
**019**	B	no	no	7/B	9	yes	ethyltoxic
**020**	C	no	no	8/B	8	no	unknown
**021**	C	no	PVT	6/A	8	no	unknown
**022**	B	no	no	6/A	7	yes	ethyltoxic

BCLC: Barcelona clinic liver cancer, MELD: Model for end-stage liver disease, PVT: portal vein thrombosis

Supplemental Table 2: Cancer staging and molecular subtype in the colorectal cancer patient cohort.

Patient ID	Biobank ID	TNM stage	Distant metastases	Tumor grade	Molecular subtype
**01**	HROC708	T3N0M0	no	/	MSI
**02**	HROC732	T3N0M0	no	G2	MSI
**03**	HROC733	T3N1M0	no	G3	MSI
**04**	HROC734	T2N2M1	yes (hepatic)	G2	MSS
**05**	HROC735	T3N1M0	no	G2	MSI
**06**	HROC736	pTis	no	/	/
**07**	HROC737	T3N0M0	no	G2	MSS
**08**	HROC739	T3N1M0	no	G3	MSS
**09**	HROC744	T3N0M0	no	G2	MSS
**010**	HROC745	T3N1M0	no	G3	MSI
**011**	HROC747	T1N0M0	no	G2	MSI
**012**	HROC749	T2N1M0	no	G2	MSS
**013**	HROC750	T3N0M0	no	G2	MSI
**014**	HROC754	T2N0M0	no	G2	MSI
**015**	HROC755	T2N0M0	no	G2	MSS
**016**	HROC759	T2N0M0	no	G2	MSS
**017**	HROC763	T3N0M0	no	G2	MSI
**018**	HROC764	T4N0M0	no	G3	MSI
**019**	HROC767	T4N0M0	no	G2	MSS
**020**	HROC770	T4N0M0	no	G1	MSS

TNM: Tumor, nodes and metastases, MSI: Microsatellite instability, MSS: Microsatellite stability

Supplemental Table 3: Classification, IDH status and MGMT methylation in the glioblastoma patient cohort

Patient ID	WHO Classification	GBM Type	IDH status	MGMT methylation
**30**	4	secondary	mutant	unmethylated
**31**	4	primary	wildtype	unmethylated
**32**	4	primary	wildtype	unmethylated
**33**	4	primary	wildtype	unmethylated
**37**	4	primary	wildtype	methylated
**38**	4	primary	wildtype	methylated
**39**	4	primary	wildtype	unmethylated
**40**	4	primary	wildtype	unmethylated
**41**	4	primary	wildtype	methylated
**44**	4	primary	wildtype	methylated
**50**	4	primary	wildtype	unmethylated

GBM: Glioblastoma, IDH: Isocitrate dehydrogenase, MGMT: O6-methylguanine-DNA methyltransferase, WHO: World Health Organization

Supplemental Table 4: Cancer staging, Combined Positive Score and p16 status in the HNSCC patient cohort

Patient ID	TNM stage	Distant metastases	Combined Positive Score	p16 status
**101**	Tx,N3,M0	no	> 1 < 5	positive
**102**	T4aN0M0	no	> 10	negative
**103**	T3N2M0	no	unkown	unkown
**104**	T3N3M0	no	unkown	negative
**105**	T4aN2bM0	no	unkown	unkown
**107**	T3N1M0	no	> 10	negative
**108**	T2N1M0	no	unkown	positive
**109**	T4N0M0	no	< 1	negative
**110**	T3N0M0	no	unkown	unkown
**111**	T4aN0M0	no	> 10	negative
**112**	T4bN2bM1	yes	> 5 < 10	negative
**113**	T4bN3M1	yes	> 1 < 5	negative
**114**	T4N0M0	no	> 10	positive
**115**	T1N3bM0	no	> 5 < 10	negative
**116**	T2N2M0	no	> 1 < 2	negative
**117**	T1N0M0	no	10.5	negative
**118**	T2N3M0	no	unkown	positive
**119**	T3N3M0	no	< 10	negative
**120**	T3N3M0	no	> 1 < 5	negative

HNSCC: Head and neck squamous cell carcinoma, TNM: Tumor, nodes and metastases

Supplemental Table 5. Antibodies used for spectral flow cytometry

Target	Fluorophore	Supplier	Order no.
PD1	BV421	BioLegend	367422
Live-Dead	FVS450	ebioscience	65-0863-14
CD45RO	BV480	BD Bioscience	566143
CD14	BV510	BioLegend	367124
CD8	BV570	BioLegend	301038
CD19	BV605	BioLegend	302244
CD16	BV650	BioLegend	302042
CD56	BV711	BioLegend	362542
CD4	BV750	BioLegend	344644
CD11c	BV785	BioLegend	301644
CD142	FITC, REAfinity™	Miltenyi Biotec	130-115-683
CD3	Spark Blue 550	BioLegend	344852
CD25	PE	Miltenyi Biotec	130-113-286
FOXP3	PE/Dazzle594	BioLegend	320126
CD95	PE/Cy5	BioLegend	305610
CD163	PerCP/Cy5.5	BioLegend	326512
CD87	PerCP-eFluor™ 710	eBioscience	46-3879-42
CD177	PE-Vio770	Miltenyi Biotec	130-101-527
CD206	APC	BioLegend	321110
ACE2	AF647	RnD Systems	FAB9332R-100UG
CD20	Spark NIR 685	BioLegend	302366
CD127	APC-R700	BD Bioscience	565185
CD45RA	APC/Fire750	BioLegend	304152
CD11b	APC/Fire810	BioLegend	302272
